# Probiotic Properties In Vitro of *Bacillus velezensis* FJAT-57093 with Antibacterial Activity Against the Aquatic Pathogen *Aeromonas hydrophila*

**DOI:** 10.3390/microorganisms14010041

**Published:** 2025-12-23

**Authors:** Yanping Chen, Suyi Li, Wenjie Li, Xuefang Zheng, Meichun Chen, Xin Liu, Jianglin Lan, Jieping Wang

**Affiliations:** 1 Institute of Resources, Environment and Soil Fertilizer, Fujian Academy of Agricultural Sciences, Fuzhou 350003, China; chenyanping@faas.cn (Y.C.); 13859315255@163.com (W.L.); zhengxuefangfz@163.com (X.Z.); cmczjw@163.com (M.C.); fzliu@yeah.net (X.L.); 2 Institute of Biotechnology, Fujian Academy of Agricultural Sciences, Fuzhou 350003, China; lisuyi@faas.cn

**Keywords:** *Bacillus velezensis*, *Aeromonas hydrophila*, aquatic probiotics, probiotic properties, safety assessment

## Abstract

The aim of this study was to acquire endospore-former(s) for aquatic animal feed based on the probiotic potential in vitro, including the anti-pathogen spectrum, gastrointestinal fluid tolerance, antioxidant activity, enzyme-producing ability, and basic safety assessment. The strain *Bacillus velezensis* FJAT-57093 was found to exhibit the strongest antibacterial ability against *Aeromonas hydrophila* in the agar well diffusion inhibition assays from 111 *Bacillus*-like strains. Moreover, the results showed that the compounds of the strain FJAT-57093 enriched by acid precipitation might be the main antibacterial metabolites. The strain FJAT-57093 also exhibited antibacterial effects against the aquatic pathogens *Photobacterium damselae*, *Edwardsiella tarda*, *Vibrio parahaemolyticus*, and *Vibrio vulnificus*. The safety assessment revealed that the strain FJAT-57093 was non-hemolytic and susceptible to ten antibiotics. The putative virulence and antibiotic resistance genes predicted were predominantly intrinsic to the FJAT-57093 genome. Furthermore, the strain FJAT-57093 demonstrated a tolerance of acid and bile salt under the simulated gastrointestinal tract conditions, extracellular enzyme-producing abilities, as well as an auto-aggregation rate of 45.88% at 24 h and co-aggregation rates with the aforementioned five aquatic pathogens, ranging from 14.87% to 58.55%. Additionally, its extracellular metabolites displayed strong antioxidant activities, with ABTS^+^ and DPPH radical scavenging rates of up to 99.82% and 42.74%, respectively. In summary, the strain *B. velezensis* FJAT-57093 was found to possess strong antibacterial activities against multiple aquatic pathogens and desirable in vitro probiotic properties.

## 1. Introduction

Aquatic animals such as fish, shrimp, and shellfish can provide high-quality proteins and various bioavailable micronutrients and are increasingly crucial for global food security [1]. However, bacterial diseases are a common challenge in aquaculture, leading to massive mortality and economic losses. *Aeromonas hydrophila* is a Gram-negative, facultatively anaerobic, and common pathogen that causes gastroenteritis and wound infections in aquatic animals (particularly fish) [2,3]. Moreover, outbreak of *A. hydrophila* infection becomes more frequent and severe with the expansion of high-density aquatic farming. On the other hand, its broad host range and diverse virulence factors complicate disease control [4,5]. Currently, antimicrobials are widely used in aquaculture to prevent and treat *A. hydrophila* infections. However, the intensive use of antibiotics often results in drug residues, global spread of antibiotic resistance genes (ARGs), and the rise of antibiotic-resistant bacteria (ARB), causing considerable risks to aquatic ecosystems and public health [6,7]. Therefore, an effective and green method for *A. hydrophila* control is urgently required.

One of the preventive measures for infectious disease control is the use of probiotics in aquatic feeds, due to their high efficacy, safety, ease of use, and environmental friendliness [8]. Other than suppressing pathogens, the use of probiotics in aquaculture can also increase nutrient utilization and feed conversion, modulate the host immune system, improve water quality, and thus promote overall health and growth of the farmed animals, thereby offering a multi-pronged solution [9,10,11]. Among the promising probiotics, the *Bacillus*-like bacteria that can form endospores are increasingly utilized in aquaculture because of their robust stress tolerance, versatile secondary metabolites, and diverse modes of action [12]. Moreover, the most frequently used *Bacillus*-like species in aquaculture were *Bacillus subtilis*, *Bacillus velezensis*, *Bacillus amyloliquefaciens*, and *Heyndrickxia coagulans* (formerly *Bacillus coagulans*) [13,14,15,16,17].

With the increasing consumer demand for high-quality aquatic products and the growing disease issues in aquaculture, probiotics have emerged as the preferred alternative to antibiotics and chemical agents for pathogen control. In this study, *A. hydrophila* FJAT-52715 was used as an indicator pathogen to screen probiotic candidates with antibacterial activity from 111 *Bacillus*-like strains. A series of in vitro experiments were conducted to systematically evaluate their probiotic potential, including hemolytic activity, virulence gene detection, antibiotic susceptibility, prediction of putative virulence factors and ARGs, antibacterial spectrum, tolerance to bile salts and simulated gastrointestinal fluids, auto-aggregation and co-aggregation capabilities, antioxidant activity, and digestive enzyme production. Our work thereby provides probiotic candidate(s) with comprehensive potential for aquaculture.

## 2. Materials and Methods

### 2.1. Strains, Culture Conditions, and Reagents

*Pathogenic bacteria*. Seven aquaculture pathogenic bacteria were used in this study. *Aeromonas hydrophila* FJAT-52715, *Edwardsiella tarda* FJAT-52713, *Photobacterium damselae* FJAT-52714, and *Vibrio vulnificus* FJAT-52716 were isolated from infected fish bodies by the Institute of Biotechnology, Fujian Academy of Agricultural Sciences (Fuzhou, China). *Vibrio parahaemolyticus* FJAT-2503, *Vibrio harveyi* FJAT-2510, and *Vibrio alginolyticus* FJAT-2512 were provided by Xiamen Changke Bio-engineering Co., Ltd. (Xiamen, China), also from piscine origins. *Staphylococcus aureus* FJAT-2450 was used as a positive control in the hemolytic activity assay and preserved in the Fujian Bacilli Resource Collection Center (FBRCC), Institute of Resources, Environment and Soil Fertilizer, Fujian Academy of Agricultural Sciences.

*Bacillus-like bacteria*. A total of 111 *Bacillus*-like strains were used to screen probiotic candidates with antibacterial activities against *A. hydrophila* (Table S1), which were preserved in the FBRCC.

*Culture conditions*. The aquatic pathogenic and *Bacillus*-like bacteria and *S. aureus* could all grow in Luria–Bertani (LB) media (10 g tryptone, 5 g yeast extract, 10 g NaCl per 1 L distilled water, pH 7.0). Each strain was initially streaked onto LB agar plates and incubated at 30 °C for 24 h to obtain single colonies. A single colony was then inoculated in LB broth and cultured at 30 °C with shaking at 170 rpm for 24 to 48 h. The concentration of each strain was adjusted to 1 × 10^8^~1 × 10^9^ CFU·mL^−1^ for further use.

*Reagents*. Unless otherwise stated, all reagents used in this study were purchased from China National Biotec Group Co., Ltd., Beijing, China

### 2.2. Screening of Antibacterial Bacillus-like Strains Against A. hydrophila

*Primary screening*. The primary screening was performed using the spot-on-lawn assays [18]. Briefly, 100 μL cultures (1 × 10^8^ CFU·mL^−1^) of the indicator pathogen *A. hydrophila* FJAT-52715 at the mid-log phase were evenly spread onto the LB agar plates. After the surface drying, 2.0 μL cultures (1 × 10^8^ CFU·mL^−1^) of each *Bacillus*-like strain were spotted onto the inoculated agar plates. At the same time, 80 μL of ciprofloxacin (0.064 µg·mL^−1^; TartetMol, Co., Ltd., Shanghai, China; Cat. No. T1640) and fresh LB broth were used as the positive and negative controls, respectively. The plates were then incubated statically at 30 °C for 48 h. The formation and size of inhibition zones were observed and measured at 24 h and 48 h, respectively.

*Secondary screening*. The *Bacillus*-like strains that showed antibacterial activity in the primary screening were further evaluated using the agar well diffusion assays [19]. Briefly, 6 mm diameter wells were aseptically punched into the agar spread with 100 μL cultures (1 × 10^8^ CFU·mL^−1^) of *A. hydrophila* FJAT-52715, and each well was filled with 80 μL cultures (1 × 10^8^ CFU·mL^−1^) of one *Bacillus*-like candidate. The positive and negative controls were set as mentioned above. After static incubation at 30 °C for 48 h, the diameters of the inhibition zones were measured. Ultimately, the *Bacillus velezensis* FJAT-57093 with the strongest antibacterial activity against *A. hydrophila* was selected for further studies

### 2.3. Genome Sequencing and Analyses of B. velezensis FJAT-57093

The genome sequencing, assembly, and annotation were all performed by Shanghai Majorbio Biopharmaceutical Technology Co., Ltd. (Shanghai, China). Briefly, genomic DNA of *B. velezensis* FJAT-57093 was extracted from the cultures grown at 30 °C in LB broth for 24 h using the Promega Wizard Genomic DNA Purification Kit (Madison, WI, USA). DNA quality was assessed by 1% agarose-gel electrophoresis, Qubit fluorometry, and NanoDrop (Thermo Fisher Scientific, Waltham, MA, USA) spectrophotometry, respectively. Both PacBio RS II and Illumina NovaSeq X Plus (Illumina, San Diego, CA, USA) platforms were used to sequence the genome of *B. velezensis* FJAT-57093. Hybrid reads were assembled de novo using Unicycler (SPAdes v 4.0.0).

The genomic analyses were performed through the online tools of the Majorbio Cloud Platform (https://www.majorbio.com/tools, accessed on 19 December 2025) [20]. The average nucleotide identity (ANI) was calculated using the Ortho ANIu algorithm [21]. An ANI value cut-off of 96% is recommended for species delineation [22]. Then, gene prediction and functional annotation of *B. velezensis* FJAT-57093 were performed with Glimmer(v3.0), followed by BLAST (v2.3.0) searches against the NCBI Non-Redundant Protein Database (NR), Swiss-Prot (EBI, European bioinformatics institute), Clusters of Orthologous Groups of proteins (COG), Gene Ontology (GO), and Kyoto Encyclopedia of Genes and Genomes (KEGG). Putative virulence factors and AGRs were predicted by using the Virulence Factor Database (VFDB) [23] and the Comprehensive Antibiotic Resistance Database (CARD) [24], respectively. To evaluate whether the putative virulence factors are typical, low-risk housekeeping, or high-risk features, a pangenome analysis of 434 *B. velezensis* genomes (including all NCBI complete genomes and FJAT 57093) was performed using PGAP2(v1.0.6)  (https://github.com/bucongfan/PGAP2, accessed on 19 December 2025) [25] to map virulence genes of FJAT 57093 to pangenome categories. To assess the potential mobility of the putative AGRs, mobile genetic elements (plasmids, prophages, insertion sequences, inverted repeat elements, and compositional outlier regions) were searched in the genome using the Mobilome Annotation Pipeline (v4.2.0) developed by EBI-Metagenomics (https://github.com/EBI-Metagenomics/mobilome-annotation-pipeline, accessed on 19 December 2025). Biosynthetic gene clusters potentially involved in antimicrobial metabolite production were mined using antiSMASH 7.0 [26].

### 2.4. Extraction and Activity Assessment of FJAT-57093 Antibacterial Compounds

Firstly, the cell-free supernatants of FJAT-57093 were acquired from the cultures in LB liquid medium at 30 °C with shaking at 170 rpm for 48 h by centrifuging at 4000× *g* for 10 min. Secondly, these supernatants were acidified to pH 2.0 using 3 M HCl to precipitate antibacterial compounds. The precipitate was collected, re-dissolved in phosphate-buffered saline (PBS) solution (pH 6.8), lyophilized, and stored as a dry powder [27]. Finally, the resulting powder was dissolved in sterile ddH_2_O to prepare the antibacterial compound solution for antimicrobial activity testing.

Activity of FJAT-57093 antibacterial compounds against *A. hydrophila* FJAT-52715 was evaluated using the agar well diffusion method as described in the Section 2.2. In each 6 mm well, 80 µL of antibacterial compound solution (40 mg·mL^−1^), cell-free supernatant, bacterial cell suspension, or fermentation broth was separately added. Fresh LB broth and ciprofloxacin (0.064 µg·mL^−1^) were used as the negative and positive controls, respectively. The plates were incubated at 30 °C for 48 h, and the diameters of inhibition zones were measured.

### 2.5. Hemolytic Activity Assay

The hemolytic activity of FJAT-57093 was evaluated as previously described [28]. Briefly, 1 μL of a mid-exponential phase culture (1 × 10^8^ CFU·mL^−1^) was spotted onto the Columbia blood agar plate (Shanghai Shenqi Bio-Technology Co., Ltd., Shanghai, China), using *S. aureus* FJAT-2450 and LB broth as a positive and negative controls, respectively. After incubation at 30 °C for 24 h, the plates were inspected for hemolysis. Hemolytic patterns were categorized as follows: α-hemolysis (greenish zone around the colony), β-hemolysis (clear, transparent zone), or γ-hemolysis (no zone, indicating no hemolysis).

### 2.6. Antibiotic Susceptibility Tests

The antibiotic susceptibility profile of the strain FJAT-57093 was determined using the Kirby–Bauer disk diffusion method. A Mueller–Hinton broth (MHB) agar plate was coated with 100 μL of logarithmic-phase cultures (1 × 10^8^ CFU·mL^−1^). After the surface drying, the antibiotic disks (Hangzhou Microbial Reagent Co., Ltd., Hangzhou, China) were aseptically placed onto the agar. The 13 tested antibiotics disks were cefaclor (30 μg), erythromycin (15 μg), chloramphenicol (30 μg), enrofloxacin (10 μg), gentamicin (10 μg), doxycycline (30 μg), neomycin (30 μg), kanamycin (30 μg), tetracycline (30 μg), polymyxin B (300 μg), streptomycin (10 μg), penicillin G (10 μg), and ampicillin (10 μg). The plates were incubated at 30 °C for 24 h, and then the inhibition zone diameters were measured and the antibiotic susceptibility was interpreted according to “Performance Standards for Antimicrobial Susceptibility Testing, the 45th Edition” of the Clinical and Laboratory Standards Institute (CLSI).

The minimum inhibitory concentrations (MICs) of antibiotics, to which the strain FJAT-57093 was resistant according to the susceptibility tests, were determined using the broth microdilution method recommended by CLSI. Cultures of the strain FJAT-57093 in the logarithmic growth phase were diluted with MHB to prepare working solutions with a concentration of approximately 5 × 10^5^ CFU·mL^−1^. The test antibiotics were serially twofold diluted to a concentration range of 0.00625 to 12.8 µg·mL^−1^ in 96-well plates. Subsequently, an equal volume of the FJAT-57093 working solution was added to each well, resulting in a final bacterial concentration of approximately 2.5 × 10^5^ CFU/mL per well (ODs). A negative control (the FJAT-57093 working solution and MHB broth were mixed at equal volumes without any antibiotic, ODn) and blank controls (MHB broth alone, ODb) were included in the plates. After sealing, the plates were incubated at 30 °C for 24 h, and the absorbance at 600 nm (OD_600 nm_) was measured using a microplate reader. The inhibition rate (IA) was calculated using the following formula: IA (%) = [(ODn − ODb) − (ODm- ODb)]/(ODn − ODb) × 100.

### 2.7. Anti-Pathogen Spectrum Tests

The antibacterial activities of the strain FJAT-57093 against the aquatic pathogens *E. tarda* FJAT-52713, *P. damselae* FJAT-52714, *V. vulnificus* FJAT-52716, *V. parahaemolyticus* FJAT-2503, *V. harveyi* FJAT-2510, and *V. alginolyticus* FJAT-2512 were evaluated using the agar well diffusion method as described in the section “*Secondary screening*”. The antibacterial abilities were determined by measuring the diameters of the inhibition zones. At the same time, 80 μL each of ciprofloxacin (0.25 µg·mL^−1^ for FJAT-52713, FJAT-52714 and FJAT-52716; and 1.5 µg·mL^−1^ for FJAT-2503, FJAT-2510, and FJAT-2512) and LB broth were used as the positive and negative controls, respectively.

### 2.8. Assessment of Bile Salt Tolerance

The bile salt tolerance of strain FJAT-57093 was evaluated according to the methods described by Argyri et al. [29], Ahire et al. [30], and Jang et al. [31] with slight modifications. Briefly, 5 mL of logarithmic-phase cultures (1 × 10^8^~1 × 10^9^ CFU·mL^−1^) was harvested by centrifugation and resuspended in an equal volume of LB broth containing 0.3% and 0.5% (*w*/*v*) bile salts, respectively. These suspensions were incubated at 30 °C with shaking at 170 r/min for 3 h. Finally, the viable bacterial count of each sample was determined using the spread plate method. The survival rate (Ms) of the strain was calculated using the following formula:

(1) Ms (%) = (As/A_0_) × 100%

where A_0_ represents the initial viable bacterial count in the cultures of the strain FJAT-57093 before centrifugation, and As represents the viable bacterial count after 3 h of bile salt treatment.

### 2.9. Assessment of Gastric and Intestinal Fluid Tolerance

The gastric and intestinal fluid tolerance of the strain FJAT-57093 was evaluated according to methods described by Guo et al. [32] and James et al. [15] with slight modifications. The simulated gastric and intestinal fluids were prepared as sterile phosphate-buffered saline (PBS) solution (pH 2.0, 3.0, and 4.0) containing 1 g/dL pepsin (Solarbio Co., Ltd., Beijing, China; 1:3000; Cat. No. P8390), and as PBS solution (pH 6.8) containing 1 g/dL pancreatin (Solarbio Co., Ltd., Beijing, China; 1:250; Cat. No. T8150), respectively. All solutions were filter-sterilized using a 0.22 µm membrane (Shanghai Xingshang Filtration Technology Co., Ltd., Beijing, China).

Firstly, cells of FJAT-57093 were harvested from 5 mL cultures of FJAT-57093 in the logarithmic phase (OD_600 nm_ ≈ 1.5) by centrifugation at 4000× *g* for 10 min and resuspended in an equal volume (5 mL) of simulated gastric fluid at each tested pH, and the suspensions were then incubated at 30 °C with shaking at 170 rpm. Aliquots were taken at 3 h for the determination of viable cell counts using the spread plate method. After the 3 h simulated gastric fluid treatment phase, the remaining cell suspensions were transferred into the simulated intestinal fluids, respectively, and incubated for another 3 h under the same conditions. An aliquot sample was taken at the end of this simulated intestinal fluid treatment phase for viable cell counting. The survival rates after gastric (Mg) and intestinal (Mi) treatments were separately calculated using the following formulas:

(2) Mg (%) = (Ag/A_0_) × 100%

(3) Mi (%) = (Ai/A_0_) × 100%

where A_0_ is the initial viable bacterial count in the cultures of the strain FJAT-57093 before centrifugation, Ag is the viable count after 3 h in gastric fluid, and Ai is the viable count after the subsequent 3 h in intestinal juice.

### 2.10. Determination of Aggregation Ability

The auto-aggregation and co-aggregation abilities of the strain FJAT-57093 were evaluated according to methods described by Gupta et al. [33] and Bao et al. [34]. The strain FJAT-57093 and five pathogens determined by anti-pathogen spectrum testing were cultured in LB broth at 30 °C with shaking at 180 rpm for 48 h. Cells of each strain were harvested by centrifugation and resuspended in sterile PBS (pH 6.8) to a final concentration of approximately 1 × 10^8^ CFU·mL^−1^.

*Auto-aggregation Assay*. The resuspended cells of FJAT-57093 were vortexed, aliquoted into a 96-well plate, and incubated statically at 30 °C for 24 h. The OD_600 nm_ values were measured immediately (ODi) and after 24 h (Todt), respectively. The auto-aggregation percentage (AA) was calculated as follows: AA (%) = [(ODi − ODt)/ODi] × 100.

*Co-aggregation Assay*. Co-aggregation was assessed by mixing the suspension of FJAT-57093 with each pathogenic suspension at a 1:1 (*v*/*v*) ratio. The mixtures were vortexed and incubated statically at 30 °C for 24 h. The OD_600 nm_ values were measured for the individual suspensions of FJAT-57093 (ODb) and each pathogen (ODp) before mixing with each other, and for the mixture after 24 h (ODm), respectively. The co-aggregation percentage (CA) was calculated using the formula CA (%) = {[(ODb + ODp)/2] − ODm}/[(ODb + ODp)/2] × 100.

### 2.11. Determination of Antioxidant Activity

Firstly, a culture of the strain FJAT-57093 was adjusted to a concentration of 7.2 × 10^8^ CFU·mL^−1^. Secondly, the resulting bacterial liquid underwent doubling dilution using PBS (pH 6.8) to obtain serial concentration bacterial liquids of 36, 18, 9, 4.5, and 2.25 × 10^7^ CFU·mL^−1^, respectively. Thirdly, the 6 serial concentration bacterial liquids were centrifuged at 4000× *g* for 10 min to harvest bacterial cell pellets and fermentation supernatants, respectively, and then the cell pellets were resuspended in equal volumes of sterile PBS (pH 6.8) to create cell suspensions. Finally, 6 cell suspensions and 6 fermentation supernatants were used to determine their ABTS^+^ and DPPH radical scavenging activities, respectively. The ABTS^+^ and DPPH radical scavenging activities were determined according to the established methods [35,36], and vitamin C was used as a positive control.

*ABTS^+^ Radical Scavenging Assay*. A stock solution was prepared by mixing 7 mmol/L ABTS^+^ solution (Yuanye Bio Co., Ltd., Shanghai, China; Cat. No. S19198) with an equal volume of 2.45 mmol/L K_2_S_2_O_8_ solution, following a reaction in the dark at 30 °C for 12 h. The resulted solution was diluted with 70% ethanol to an absorbance of 0.70 at 734 nm to obtain the working solution. Subsequently, 29 μL of the FJAT-57093 cell suspension and fermentation supernatant was mixed with 171 μL of the working solution in a 96-well plate, respectively. After incubation in the dark at 25 °C for 10 min, the absorbance at 734 nm (OD_sample) was measured. The sample background (OD_sample_blank) was determined by mixing the FJAT-57093 cell suspension and fermentation supernatant with 70% ethanol, but without ABTS^+^ working solution. Meanwhile, the reagent blank (OD_reagent_blank) was measured using 70% ethanol mixed with the ABTS^+^ working solution, but without the tested bacterial cell suspension and fermentation supernatant samples. The scavenging rate was calculated as follows: ABTS^+^ Scavenging Rate (%) = [1 − (OD_sample − OD_sample_blank)/OD_reagent_blank] × 100.

*DPPH Radical Scavenging Assay*. The 0.1 mmol/L DPPH (Yuanye Bio Co., Ltd., Shanghai, China; Cat. No. S30629) solution was prepared in absolute ethanol and stored in the dark. Then, 0.1 mL each of the FJAT-57093 bacterial cell bacterial suspension and fermentation supernatant were mixed with 0.1 mL of the DPPH solution, respectively. The mixture was incubated at 30 °C in the dark for 30 min, and then the absorbance was measured at 520 nm (OD_sample). A mixture of 0.1 mL absolute ethanol and 0.1 mL DPPH solution served as the blank control (OD_control). The scavenging percentage was calculated as follows: DPPH Scavenging Percentage (%) = (OD_control − OD_sample)/OD_control × 100.

### 2.12. Determination of Digestive Enzyme-Producing Ability

The extracellular amylase, protease, and cellulase activities of the strain FJAT-57093 were evaluated using plate-based assays as described by Uyar et al. [37], Nair et al. [38], and Nashad et al. [39], respectively. Briefly, 2 µL cultures (1 × 10^8^ CFU·mL^−1^) of FJAT-57093 at the logarithmic phase were spotted onto the center of starch agar (3 g beef extract, 10 g peptone, 20 g soluble starch, 20 g agar per 1 L distilled water, pH 7.0), skim milk agar (3 g beef extract, 10 g peptone, 50 g skim milk powder, 20 g agar per 1 L distilled water, pH 7.0), and cellulose agar (10 g sodium carboxymethyl cellulose (CMC-Na), 5 g yeast extract, 5 g peptone, 5 g NaCl, 20 g agar per 1 L distilled water, pH 7.0) plates, respectively. The plates were incubated statically at 30 °C for 24 h. The production ability of 3 extracellular digestive enzymes was non-quantitatively assessed according to the clear hydrolysis zone on the corresponding agar plate.

### 2.13. Data Analysis

All experiments were performed with three independent replicates. Data were analyzed by one-way ANOVA followed by Duncan’s new multiple range test (Duncan) using DPS software (v21.05; Hangzhou Ruifeng Information Technology Co., Ltd., Hangzhou, China). Results are presented as the mean ± standard deviation, with statistical significance defined as *p* < 0.05.

## 3. Results

### 3.1. Screening of Probiotic Candidates with Antibacterial Activities Against A. hydrophila

**Figure 1:**
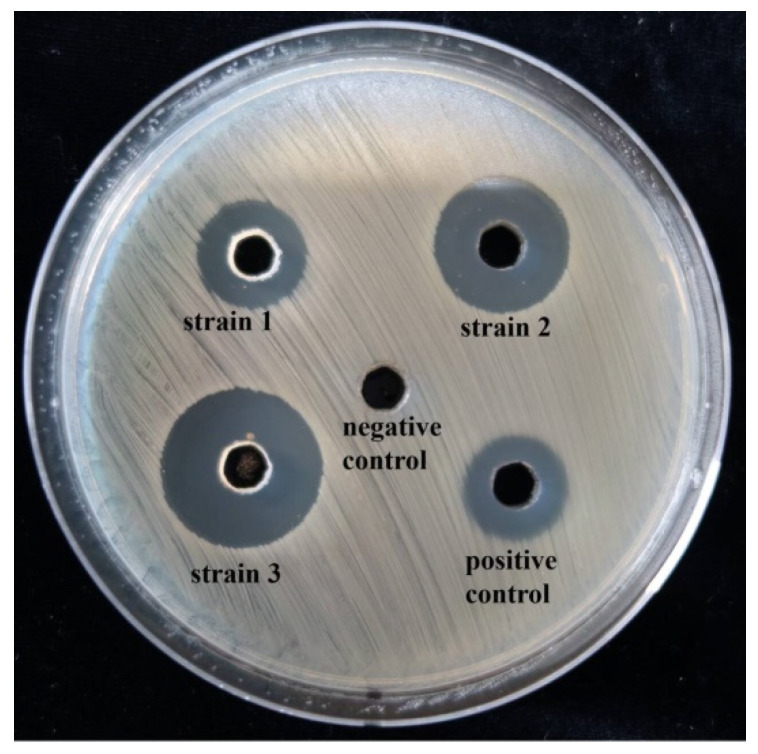
Screening of antibacterial *Bacillus*-like bacteria against *Aeromonas hydrophila*. The *Bacillus*-like strains that showed antibacterial activity in the primary screening were further evaluated using the agar well diffusion assays. Ciprofloxacin (0.064 µg·mL^−1^) and fresh LB broth were used as the positive and negative controls, respectively. The strains 1, 2, and 3 were *B. velezensis* FJAT-57117, *B. velezensis* FJAT-17931, and *B. velezensis* FJAT-57093, respectively.

A total of 111 *Bacillus*-like isolates (Table S1) were used to screen antibacterial strains against *A. hydrophila*. The results revealed that 16 strains exhibited remarkably antibacterial activities with inhibition zone diameters from 10.42 to 20.83 mm (Table 1). Among them, the strain FJAT-57093 displayed the strongest antibacterial activity against *A. hydrophila* (Figure 1). Although the taxonomic status had been validated previously by 16S rRNA and *gyrB* sequence analyses, taxonomic identification of the strain FJAT-57093 was further conducted by the whole-genome ANI analysis. As shown in Figure S1, the ANI values of the strain FJAT-57093 with the strains *B. velezensis* FZB42, AP46, and CBMB205 were all higher than 97.7%, which exceeded the recommended cut-off value of 96% ANI for species delineation [22], while those with the strains *B. cereus* MLLY1 and P1, *B. subtilis* 168 and MCIB3610, and *B. thuringiensis* ATCC0792 were all lower than 85.5%. Thus, the strain FJAT-57093 was confirmed again to be a member of the species *B. velezensis*, and the strain *B. velezensis* FJAT-57093 was selected for further studies.

### 3.2. Genomic Analyses of the Strain B. velezensis FJAT-57093

Generally, the genome of the strain FJAT-57093 comprised a circular chromosome of 3,452,556 bp, with an average G + C content of 46.49%, and no plasmid was discovered (Figure S2). A total of 3730 protein-coding genes were predicted. The genome sequence data of the strain FJAT-57093 were deposited in GenBank under the accession number PRJNA1364769.

To screen the putative virulence and ARG-related genes, the *B. velezensis* FJAT-57093 genome underwent searches against the databases VFDB and CARD, respectively. The results showed that the putative virulence factors predicted in *B. velezensis* FJAT-57093 were associated with functions such as adherence, effector delivery system, exoenzyme, exotoxin synthesis, immune modulation, invasion, and stress survival (Table S2). Additionally, 38 categories of putative ARGs were identified in *B. velezensis* FJAT-57093, which were primarily related to aminoglycoside, carbapenem, cephalosporin, fluoroquinolone, glycopeptide, macrolide, penam, peptide, phenicol, rifamycin, and tetracycline (Table S3).

The results of mobilome annotation revealed that 499 (96.9%) of the identified 515 putative resistance and virulence genes showed no evidence of mobile genetic elements within their flanking 100 bp regions (Table S4), suggesting that these putative virulence factors and ARGs were predominantly intrinsic to the FJAT-57093 genome. The pangenome analysis of 434 *B. velezensis* genomes using PGAP2 revealed that the species *B. velezensis* exhibited an open pangenome with total genes of 27,784, and 84.00% cloud genes, 5.26% shell genes, and only 1.48% strict core genes (Table S5). Moreover, the putative virulence genes of FJAT 57093 displayed a pangenome category of 62.8% cloud genes, 16.0% core genes, 8.2% shell genes, 3.6% soft core genes, and 9.4% strict core genes (Figure S3). This pangenome category indicated that most VFDB annotations of FJAT 57093 virulence genes represented strain-specific accessory functions rather than conserved pathogenic determinants.

**Figure 2:**
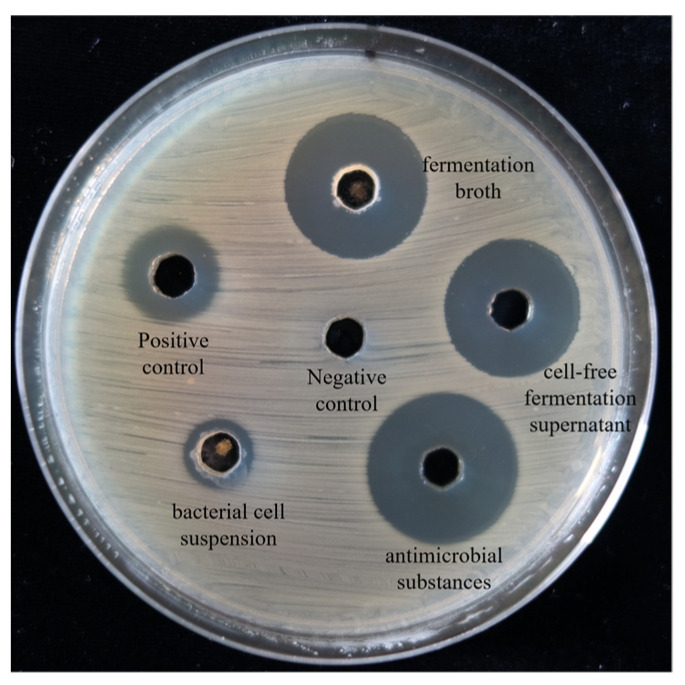
Identification of the antimicrobial substances from *B. velezensis* FJAT-57093. The antibacterial compounds were extracted from culture supernatants of the strain FJAT-57093 by the acid (pH 2.0 using 3 M HCl) precipitation method. Activity of the FJAT-57093 antibacterial compounds against *A. hydrophila* was evaluated using the agar well diffusion method. Fresh LB broth and ciprofloxacin (0.064 µg·mL^−1^) were used as the negative and positive controls, respectively.

Furthermore, the biosynthetic gene clusters (BGCs) potentially associated with the antibacterial metabolites were predicted by using antiSMASH 7.0. A total of 17 BGCs were identified in the FJAT-57093 genome, which were mainly related to biosynthesis of antibacterial compounds bacillaene, bacilysin, difffcidin, fengycin, lichenysin, macrolactin, plipastatin, and surfactin (Table S6). According to the results of BGC prediction, we attempted to extract the antibacterial compounds by acid precipitation from the cultures of the strain FJAT-57093 and evaluate their antibacterial activity against *A. hydrophila* using the agar well diffusion method. As shown in Figure 2, antibacterial activity of these compounds was stronger than those of the fermentation broth and cell-free supernatant of the strain FJAT-57093, with the inhibition zone diameters of 23.97 mm, 19.58 mm, and 18.22 mm, respectively. These results indicated that the compounds enriched by acid precipitation were the primary active metabolites responsible for the antibacterial effect of this strain against *A. hydrophila*.

### 3.3. Hemolytic Activity of the Strain FJAT-57093

**Figure 3:**
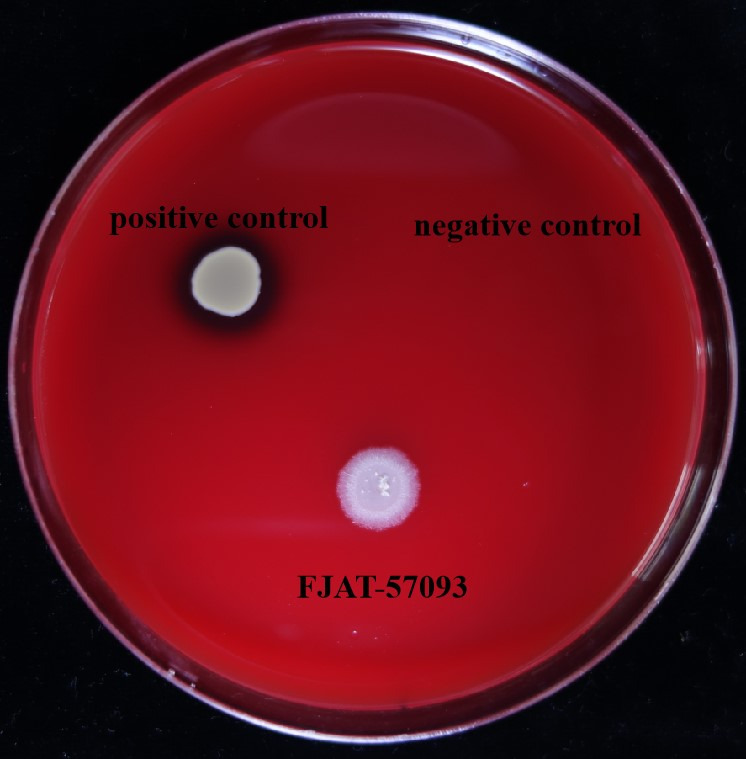
Hemolytic activity test of the strain FJAT-57093. The hemolytic activity of the strain FJAT-57093 was evaluated using the Columbia blood agar plate. *S. aureus* and fresh LB broth were used as positive and negative controls, respectively.

As shown in Figure 3, the positive control *S. aureus* FJAT-2450 exhibited typical β-hemolysis, characterizing by a clear zone around the colony. Both the strain FJAT-57093 and the negative control (fresh LB broth) showed no hemolysis, being indicative of γ-hemolysis. Therefore, the results indicated that the strain FJAT-57093 had no hemolytic activity.

### 3.4. Antibiotic Susceptibilities of the Strain FJAT-57093

Susceptibilities of the strain FJAT-57093 to 13 frequently used antibiotics were evaluated by the Kirby–Bauer disk diffusion method. The results showed that the strain FJAT-57093 exhibited high susceptibility (S) to eight antibiotics (cefaclor, erythromycin, chloramphenicol, enrofloxacin, gentamicin, doxycycline, neomycin, and kanamycin), intermediate susceptibility (I) to tetracycline and polymyxin B, and resistance (R) to three antibiotics (streptomycin, penicillin, and ampicillin) (Table 2).

**Figure 4:**
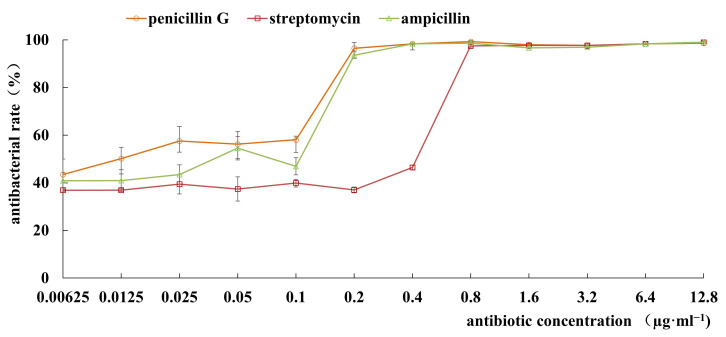
MIC determination of streptomycin, penicillin G, and ampicillin to the strain FJAT-57093. MICs of streptomycin, penicillin G, and ampicillin were determined according to “Performance Standards for Antimicrobial Susceptibility Testing, the 45th Edition” of the Clinical and Laboratory Standards Institute (CLSI). All experiments were conducted with three independent replicates.

Subsequently, the MICs of streptomycin, penicillin G, and ampicillin were determined using the broth microdilution methodology outlined by CLSI. As shown in Figure 4, the MIC values of streptomycin, penicillin G, and ampicillin were approximately 0.8 µg·mL^−1^, 0.2 µg·mL^−1^, and 0.2 µg·mL^−1^, respectively. These MIC values were all lower than the CLSI susceptibility breakpoint (≤1 µg·mL^−1^) and the relevant MIC cut-off values (4 µg·mL^−1^ for ampicillin and 8 µg·mL^−1^ for streptomycin) as recommended by the European Food Safety Authority (EFSA). Therefore, resistance of the strain FJAT-57093 to these antibiotics in disk diffusion tests could be regarded as acceptable.

### 3.5. Anti-Pathogen Spectra of the Strain FJAT-57093

In this study, seven aquatic pathogens were used to evaluate the anti-pathogen spectra of the strain *B. velezensis* FJAT-57093. As shown in Table 3, the strain FJAT-57093 exhibited antibacterial activities against *A. hydrophila*, *P. damselae*, *E. tarda*, *V. parahaemolyticus*, and *V. vulnificus*, being comparable to the corresponding positive controls. These results indicated that the strain FJAT-57093 had a broad-spectrum anti-pathogen activity.

### 3.6. Bile Salt Tolerance of the Strain FJAT-57093

**Figure 5:**
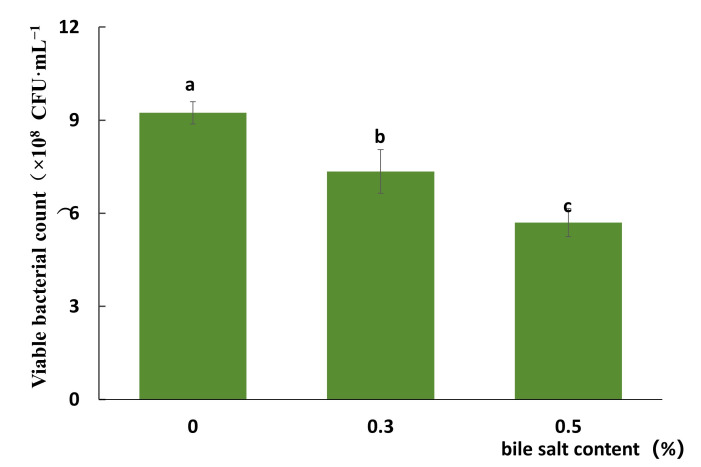
Bile salt tolerance test of the strain FJAT-57093. For bile salt tolerance test, cultures (1 × 10^8^~1 × 10^9^ CFU·mL^−1^) of the strain FJAT-57093 were incubated with LB media containing 0.3% and 0.5% (*w*/*v*) of bile salts at 30 °C with shaking at 170 r/min for 3 h, respectively. The viable bacterial count and survival rate of each sample were determined. All experiments were conducted with three independent replicates, and the different lowercase letters indicate a significant difference through the Duncan test (*p* < 0.05).

The results indicated that survival of the strain FJAT-57093 was inhibited by bile salts in a concentration-dependent manner (Figure 5). In the treatment group with 0.3% and 0.5% bile salts, the viable cell count of the strain FJAT-57093 was decreased from 9.24 × 10^8^ CFU·mL^−1^ to 7.35 × 10^8^ and 5.70 × 10^8^ CFU·mL^−1^, yielding the survival rates of 79.54% and 61.68%, respectively. These results demonstrated that the strain FJAT-57093 exhibited demonstrable tolerance to bile salt stress.

### 3.7. Gastric and Intestinal Fluid Tolerance of the Strain FJAT-57093

**Figure 6:**
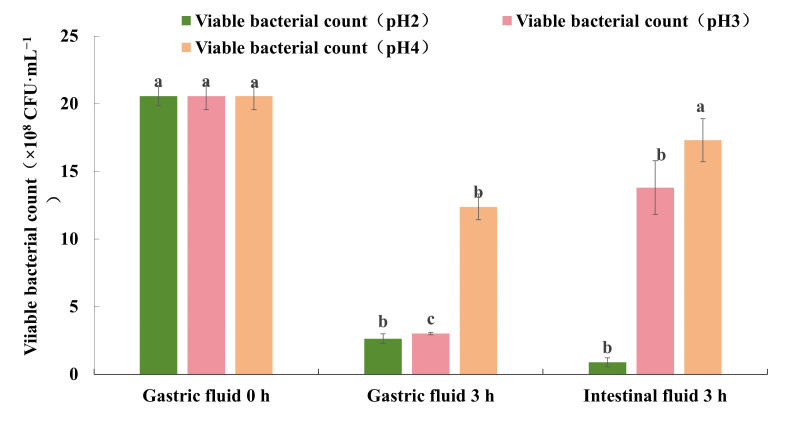
The simulated gastric and intestinal fluid tolerance tests of the strain FJAT-57093. **Notes:** For gastric and intestinal fluid tolerance test, cultures (2.06 × 10^9^ CFU·mL^−1^) of the strain FJAT-57093 successively underwent damage of the simulated gastric fluids with 1 g·dL^−1^ of pepsin at a low pH (pH 2.0, 3.0, and 4.0), and intestinal fluids (pH 6.8) with 1 g·dL^−1^ of pancreatin. All experiments were conducted with three independent replicates, and the different lowercase letters indicate significant differences of one treatment between different time points through the Duncan test (*p* < 0.05).

Except bile salt tolerance ability, the strain FJAT-57093 was further evaluated for its survival ability in the simulated gastric and intestinal fluids, in which it would undergo successively the damage of low pH, pepsin, and trypsin. The results showed that the viable cell count of the strain FJAT-57093 was significantly decreased when the strain was exposed to gastric fluids at different pH levels for 3 h (Figure 6). The survival rate of the strain FJAT-57093 was 12.77%, 14.65%, and 67.09% in the simulated gastric fluids with the pH levels of pH 2, pH 3, and pH 4, respectively. After this gastric phase, the strain FJAT-57093 was transferred to the simulated intestinal fluids (pH 6.8). Its viable cell count was decreased further in the pH 2 group, and its final survival rate was only 4.30%. In contrast, its viable cell count showed a clearly recovery-driven increase trend in the pH 3 and pH 4 groups (Figure 6), and its final survival rates increased to 67.09% and 84.12%, respectively. These results indicated that the strain FJAT-57093 was able to restore proliferation when it was transferred from an acidic pH environment (pH 3 or pH 4) in the gastric fluids to a neutral environment (pH 6.8) in the intestinal fluids, exhibiting strong tolerance to the gastrointestinal fluids. However, the strain FJAT-57093 underwent severe and irreversible damage caused by the extremely low-pH gastric environment (pH 2).

### 3.8. Aggregation Ability of the Strain FJAT-57093

**Figure 7:**
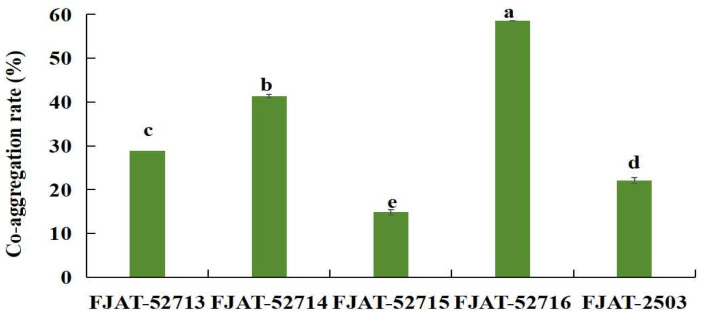
Co-aggregation of the strain FJAT-57093 with five aquatic pathogens determined by anti-pathogen spectrum testing. **Notes:** For co-aggregation ability determination, the suspensions of FJAT-57093 and each pathogen were mixed at a 1:1 (*v*/*v*) ratio and co-incubated statically at 30 °C for 24 h. The OD_600 nm_ values were measured for the individual suspension of FJAT-57093 (ODb) and each pathogen (ODp) before mixing with each other, and for the mixture after 24 h (ODm), respectively. The strains FJAT-52713, FJAT-52714, FJAT-52715, FJAT-52716, and FJAT-2503 were *Edwardsiella tarda*, *Photobacterium damselae*, *Aeromonas hydrophila*, *Vibrio vulnificus*, and *Vibrio parahaemolyticus*, respectively. All experiments were conducted with three independent replicates, and the different lowercase letters indicate a significant difference through the Duncan test (*p* < 0.05).

The auto- and co-aggregation of a potential probiotic with itself and pathogenic bacteria can facilitate intestinal colonization, thereby creating a protective film and inhibiting the adhesion of pathogens [40]. Additionally, the cited authors suggest the potential for the adsorption of pathogenic bacteria in the intestinal lumen, enabling their elimination through peristalsis. The results of this study demonstrated that the strain FJAT-57093 had an auto-aggregation rate of 45.88% after 24 h. Furthermore, the strain exhibited co-aggregation abilities with five aquatic pathogens determined by anti-pathogen spectrum testing, with a co-aggregation rate ranging from 14.87% to 58.55% (Figure 7).

### 3.9. Antioxidant Activity of the Strain FJAT-57093

**Figure 8:**
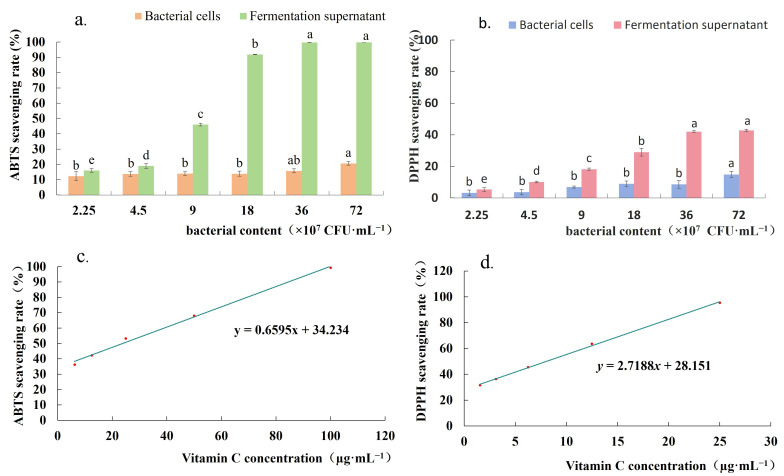
Antioxidant activity test of the strain FJAT-57093 bacterial cells and cell-free culture supernatants. (**a**,**b**) ABTS^+^ and DPPH radical scavenging capacities of the strain FJAT-57093, respectively; (**c**,**d**) the standard curve for ABTS^+^ and DPPH radical scavenging capacities of vitamin C, respectively. The cell suspensions and fermentation supernatants of serial concentration FJAT-57093 cultures (36, 18, 9, 4.5, and 2.25 × 10^7^ CFU·mL^−1^) were used to determine their ABTS^+^ and DPPH radical scavenging activities, respectively. Vitamin C was used as a positive control. All experiments were conducted with three independent replicates, and the different lowercase letters indicate a significant difference through the Duncan test (*p* < 0.05).

The antioxidant activities of the strain FJAT-57093 bacterial cells and cell-free culture supernatants were evaluated by measuring the ABTS^+^ and DPPH radical scavenging capacities, respectively. The results showed that both bacterial cells and cell-free culture supernatants exhibited ABTS^+^ and DPPH radical scavenging activities. Moreover, the ABTS^+^ and DPPH radical scavenging capacities demonstrated by the cell-free supernatants of FJAT-57093 were significantly higher than those of the bacterial cells, exhibiting an evidently concentration-dependent manner (Figure 8). The highest scavenging capacities of ABTS^+^ and DPPH radicals were only 21.25% and 14.86% by the bacterial cells, while they could reach up to 99.82% and 42.74% by the cell-free supernatants, respectively. Moreover, the highest scavenging capacities of its cell-free supernatant against ABTS^+^ and DPPH radicals were equivalent to those of 99.43 µg·mL^−1^ and 5.37 µg·mL^−1^ vitamin C, respectively. These results indicated that the extracellular metabolites of the strain FJAT-57093 were the major contributors to the observed antioxidant activities.

### 3.10. Extracellular Digestive Enzyme-Producing Ability of the Strain FJAT-57093

**Figure 9:**
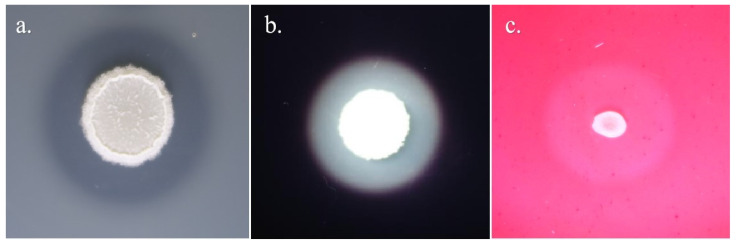
Extracellular digestive enzyme activities of the strain FJAT-57093. The production ability of (**a**) protease, (**b**) amylase, and (**c**) cellulase was non-quantitatively assessed according to the clear hydrolysis zone on the skim milk, starch, and cellulose agar plates, respectively.

Because a feed enzyme additive could improve digestibility and nutrition in animals, the strain FJAT-57093 was tested for the evaluation of its ability to produce protease, amylase, and cellulase using a clear-zone plate assay. The results confirmed that the strain FJAT-57093 could produce amylase, protease, and cellulase, and the hydrolytic zone diameters were 21.26, 16.85, and 9.23 mm (Figure 9).

## 4. Discussion

Since probiotics were first confirmed to act as biological control agents against fish diseases in the 1980s, their potential in preventing and controlling *A*. *hydrophila* infections has continued to attract research interest. Numerous studies have shown that lactic acid bacteria, *Streptomyces* spp., and *Bacillus*-like bacteria can significantly inhibit this pathogen. For instance, the *Streptomyces* strains Vitnk9 [41] and AJ8 [42] could directly suppress the growth of *A. hydrophila*; and the combinations of *Lactobacillus casei* with *B*. *subtilis* [43], *Enterococcus faecalis* MC-5 [44], and *B. methylotrophicus* WM-1 [45] could enhance host immunity, thereby improving disease resistance and survival rates.

In recent years, the endospore-forming bacterium *B. velezensis* had attracted growing attention due to its strong antibacterial activity against *A. hydrophila* and broad-spectrum antimicrobial properties. Sam-on et al. [46] isolated the strain FS26 from shrimp, which formed an inhibition zone of 23.7 mm against *A. hydrophila* and also exhibited inhibition against *A. veronii*, *V. alginolyticus*, and *V. parahaemolyticus*. Kang et al. [47] obtained the strain R-71003 from the intestine of common carp; it produced a 25.25 mm inhibition zone against *A. hydrophila* and was also effective for *A. veronii* and *Edwardsiella tarda*. Furthermore, James et al. [15] isolated the strain KM2 from mangrove sediments, which showed a 23.33 mm inhibition zone against *A. hydrophila*, along with antibacterial activities against *V. parahaemolyticus* and *Streptococcus agalactiae*. In this study, the strain *B. velezensis* FJAT-57093 produced a 20.83 mm inhibition zone against *A. hydrophila*, and its lipopeptides were preliminarily elucidated to be the primary active metabolites responsible for the antibacterial effect against *A. hydrophila*. Moreover, the strain *B. velezensis* FJAT-57093 exhibited broad-spectrum antibacterial activities against the aquatic pathogens, *P. damselae*, *E. tarda*, *V. parahaemolyticus*, and *V. vulnificus*. These results highlighted the relevance of *B. velezensis* in aquatic disease prevention, and they supported the potential of the strain FJAT-57093 as a probiotic candidate in aquaculture.

Before probiotics can be applied in aquaculture, safety validation is an essential prerequisite for commercialization. Key concerns include the risk of antibiotic resistance gene transfer and the presence of virulence factors. Many studies have shown that the ARB can transmit ARGs to other microorganisms in aquatic environments via horizontal gene transfer, facilitating the spread of antibiotic resistance, and thus undermining the effectiveness of disease prevention and posing risks to human health through the food chain [48,49]. Our results indicated that the strain *B. velezensis* FJAT-57093 was highly and intermediately sensitive to 8 and 2 of the 13 antibiotics tested, respectively. However, it is slightly regrettable that the strain FJAT-57093 has resistance to three antibiotics (streptomycin, penicillin G, and ampicillin). Therefore, MICs of streptomycin, penicillin G, and ampicillin on the strain *B. velezensis* FJAT-57093 were determined according to the guidelines of “Performance Standards for Antimicrobial Susceptibility Testing, the 45th Edition”. Fortunately, their MIC values were approximately 0.2~0.8 µg·mL^−1^ and lower than the CLSI susceptibility breakpoint (≤1 µg·mL^−1^). According to EFSA guidelines, the MIC cut-off values of ampicillin and streptomycin are 4 and 8 µg·mL^−1^ mg/L for *Bacillus* species, and penicillin G is not recommended as a key marker in the safety assessment of a probiotic *Bacillus* strain. Therefore, these results indicated that the strain FJAT-57093 could meet the requirements related to antimicrobial susceptibility testing. At the same time, putative ARGs and their flanking mobile genetic elements were further searched in the FJAT-57093 genome using CARD and the Mobilome Annotation Pipeline, respectively. The results indicated that 38 categories of putative ARGs were primarily related to aminoglycoside, carbapenem, cephalosporin, fluoroquinolone, glycopeptide, macrolide, penam, peptide, phenicol, rifamycin, and tetracycline. Only one kind of mobile genetic element, the compositional outlier region, was identified in around 7 out of 264 putative ARGs, including the antibiotic target alteration-related genes *bacA* (bacitracin) and *cdsA* (daptomycin), and antibiotic efflux-related genes *liaR* and *liaS* (daptomycin), *tetA(58)* and *mgrA* (tetracycline), and *abaF* (fosfomycin).

Studies have shown that the absence of hemolytic activity in probiotic strains generally indicates a lack of hemolysin-related toxicity [50]. Among different hemolytic phenotypes, α- and γ-hemolysis are regarded as safe, whereas β-hemolysis is considered potentially pathogenic due to its ability to release hemolysins that can damage host red blood cells [51]. In this study, *B. velezensis* FJAT-57093 exhibited a γ-hemolytic phenotype, being consistent with that of the known safe strains. Furthermore, putative virulence factors and their flanking mobile genetic elements were predicted in the FJAT-57093 genome. The results showed that the putative virulence genes predicted in the strain *B. velezensis* FJAT-57093 were mainly associated with functions such as immune modulation, metabolic factor, effector delivery system, adherence, exotoxin, and stress survival, and 11 out of 251 putative virulence genes were accompanied with one kind of mobile genetic element, the compositional outlier region. According to the pangenome analysis of 434 *B. velezensis* genomes and VFDB annotations of FJAT 57093, the potential high-risk virulence factors effector delivery systems (83.3% cloud genes), adherence factors (76.5% cloud), and exotoxins (72.7% cloud) were predominantly FJAT 57093 strain-specific and not conserved across the species *B. velezensis*, suggesting an absence of species-level pathogenic mechanisms. The core-conserved genes post-translational modification (75% core + strict core genes) and stress survival (36.4% core) were housekeeping functions for essential bacterial physiology, but not virulence mechanisms. The moderately conserved genes biofilm (34.5% core genes), motility (38.7% core), and immune modulation (27.7% core) might support probiotic activities, such as competitive exclusion, gut colonization, and immuno-stimulation, rather than pathogenicity in aquaculture. Therefore, the pangenome-contextualized analysis demonstrated that the strain FJAT57093 lacks genomic hallmarks of pathogenic bacteria. Taken together, these results suggested that the strain FJAT-57093 caused no appreciable threat for use in aquaculture, although a key in vivo safety evaluation should be addressed in the future.

Investigating the tolerance to the gastrointestinal environment of potential probiotics is a prerequisite for discerning their probiotic effects [31,52]. When the strain *B. velezensis* FJAT-57093 was transferred from a low-pH gastric acid environment (pH 3 or pH 4) to a neutral intestinal fluid environment (pH 6.8), its survival rate rapidly recovered from 14.65% and 67.09% to 67.26% and 84.12%, respectively. These results were consistent with the report by James et al. [15]: the survival rates of *B. cereus* KM1 and *B. velezensis* KM2 were 36.34% and 37% under low-pH conditions and increased to 83% and 90% after incubation in intestinal fluid at pH 8.0 for 2 h, respectively. Therefore, these results further highlighted the gastrointestinal environment adaptability of the *Bacillus*-like bacteria. On the other hand, bile salts can inhibit bacterial growth by disrupting cell membrane integrity [53]. Our results revealed that the strain FJAT-57093 could maintain a survival rate of 79.54% after 3 h of exposure to 0.3% bile salt, a concentration typically present in the intestinal environment. The abilities to withstand bile-mediated growth inhibition were also found in other *Bacillus* strains, such as *B. amyloliquefaciens* HTI-19, *B. subtilis* HTI-23, *B. cereus* KM1, and *B. velezensis* KM2, which exhibited more than 50% of survival rates after exposure to 0.3% bile salt for 3 h [15,54].

Auto-aggregation and co-aggregation abilities are key mechanisms for probiotic intestinal colonization and competitive exclusion of pathogens, representing important probiotic traits. Auto-aggregation facilitates biofilm formation, which helps strengthen the intestinal barrier [55]. Co-aggregation blocks a pathogen’s access to epithelial receptors through direct adhesion to pathogens, forming the core mechanism of competitive exclusion [56]. In this study, the strain FJAT-57093 displayed an auto-aggregation rate of 45.88% within 24 h and the co-aggregation rates ranging from 14.87% to 58.55% with the aquatic pathogens *A. hydrophila*, *P. damselae*, *E. tarda*, *V. parahaemolyticus*, and *V. vulnificus*.

Antioxidant activity is a key probiotic property that can mitigate oxidative damage in aquatic animals. The *Bacillus*-like probiotics can exert their protective effects by multiple mechanisms, including metal ion chelation, production of antioxidant enzymes, and synthesis of antioxidant metabolites [35,57,58]. Khan et al. [59] reported that *B. subtilis* and *B. licheniformis* showed DPPH radical scavenging rates of 63.80% and 53.74%, and hydrogen peroxide scavenging rates of 37.17% and 27.10%, respectively. Similarly, James et al. [15] observed that the strains *B. cereus* KM1 and *B. velezensis* KM2 exhibited DPPH scavenging rates of 60.23% and 62.53%, respectively. In this study, the fermentation supernatant of *B. velezensis* FJAT-57093 demonstrated the highest DPPH and ABTS^+^ radical scavenging rates of 42.74% and 99.82%, which were equivalent to those of 5.37 µg·mL^−1^ and 99.43 µg·mL^−1^ vitamin C, respectively. These results were consistent with the typical antioxidant profile of the *Bacillus*-like species and supported the potential application of FJAT-57093 in aquaculture.

Another notable probiotic trait is the secretion of extracellular enzymes, which promotes nutrient assimilation in aquaculture and helps to reduce the feed conversion ratio [60]. Many aquatic species lack specific digestive enzymes at their juvenile stages, so dietary supplementation with exogenous enzymes is often required to support optimal nutrition [61]. In this context, live microorganisms represent an effective source of such enzymes. For example, *B. cereus* could produce a range of extracellular enzymes and antimicrobial compounds that improve growth and immunity in *Penaeus monodon* [62]. Similarly, supplementation of *B. velezensis* could increase amylase and protease activities in mandarin fish feed, leading to an enhanced growth performance [63]. In this study, the strain FJAT-57093 could produce protease, amylase, and cellulase, which are the key extracellular enzymes involving in facilitating digestion, enhancing nutrient utilization, and supporting growth performance. These functional attributes would further strengthen the probiotic potential of the strain FJAT-57093.

## 5. Conclusions

In this study, the endospore-former *B. velezensis* FJAT-57093 was found to exhibit significant inhibitory activity against *A. hydrophila*. It was non-hemolytic and could meet the requirements related to antimicrobial susceptibility testing according to the preliminary safety assessment in vitro. The in vitro probiotic potential assessment revealed that the strain FJAT-57093 possessed good survivability throughout gastrointestinal transit, a broad spectrum of anti-pathogen and extracellular digestive-enzyme activities, good auto-aggregation and co-aggregation capabilities, and protective effects against oxidative damage. Consequently, it is inferred that the strain FJAT-57093 possesses strong antibacterial activities against multiple aquatic pathogens and desirable in vitro probiotic properties. However, there is still a long way to go to evaluate key aspects, such as the in vivo efficacy and safety, host response, and ecological impact in farming systems, to assure its suitability as a probiotic candidate in aquaculture.

## Figures and Tables

**Table 1:** The *Bacillus*-like bacteria with remarkably antibacterial activities against *Aeromonas hydrophila*.

Strain Number	Species Name ^1^	Inhibition Zone Diameter (mm) ^2^
FJAT-57093	*Bacillus velezensis*	20.83 ± 0.92
FJAT-2349	*Bacillus velezensis*	18.59 ± 0.53
FJAT-17931	*Bacillus velezensis*	17.54 ± 0.06
FJAT-57419	*Bacillus atrophaeus*	16.16 ± 0.07
FJAT-57444	*Bacillus pumilus*	15.56 ± 0.2
FJAT-57117	*Bacillus velezensis*	15.22 ± 2.28
FJAT-57108	*Bacillus thuringiensis*	15.14 ± 0.06
FJAT-57446	*Bacillus atrophaeus*	14.15 ± 0.15
FJAT-57104	*Bacillus rugosus*	13.46 ± 0.4
FJAT-57440	*Bacillus haynesii*	13.08 ± 0.3
FJAT-57102	*Bacillus swezeyi*	12.44 ± 0.92
FJAT-57417	*Bacillus pumilus*	12.17 ± 0.69
FJAT-57439	*Bacillus velezensis*	11.32 ± 0.36
FJAT-57100	*Bacillus zhangzhouensis*	10.97 ± 0.03
FJAT-57118	*Bacillus thuringiensis*	10.58 ± 0.28
FJAT-46513	*Bacillus amyloliquefacien*	10.42 ± 0

**Notes:** ^1^ The taxonomic information of each strain had been validated by 16S rRNA and gyrB sequence analyses. ^2^ All experiments were conducted with three independent replicates, and the data were presented as the mean ± standard deviation.

**Table 2:** Antibiotic sensitivity test of the strain FJAT-57093.

Antibiotics	Content per Tablet/μg	Judgement Standard/mm ^1^			Inhibition Zone Diameter ^2^	Sensitivity
		Drug Tolerance (R)	Inhibition (I)	High Sensitivity (S)		
cefaclor	30	≦14	15–17	≧18	35.21 ± 0.45	S
erythromycin	15	≦13	14–22	≧23	31.99 ± 0.77	S
chloramphenicol	30	≦12	13–17	≧18	29.71 ± 0.77	S
enrofloxacin	10	≦22	23–27	≧28	28.15 ± 0.65	S
gentamicin	10	≦12	13–14	≧15	25.61 ± 0.58	S
doxycycline	30	≦12	13–15	≧16	23.92 ± 0.75	S
neomycin	30	≦12	13–16	≧17	22.28 ± 0.60	S
kanamycin	30	≦13	14–17	≧18	20.03 ± 0.85	S
tetracycline	30	≦14	15–18	≧19	17.43 ± 0.18	I
polymyxin B	300	≦8	8–11	≧12	8.40 ± 0.06	I
streptomycin	10	≦11	12–14	≧15	8.76 ± 0.27	R
penicillin G	10	≦19	20–27	≧28	0.00 ± 0.00	R
ampicillin	10	≦13	14–16	≧17	0.00 ± 0.00	R

**Notes:** ^1^ The breakpoint criteria in antibiotic susceptibility testing were defined according to “Performance Standards for Antimicrobial Susceptibility Testing, the 45th Edition” of the Clinical and Laboratory Standards Institute (CLSI). ^2^ All experiments were conducted with three independent replicates. Data were presented as the mean ± standard deviation.

**Table 3:** Antibacterial activity assay of the strain FJAT-57093 against aquatic pathogens.

Strain Number	Species Name	Inhibition Zone Diameter of Fermentation Broth (mm)	Inhibition Zone Diameter of Positive Control (mm)
FJAT-52715	*Aeromonas hydrophila*	20.83 ± 0.92	13.72 ± 0.18
FJAT-52714	*Photobacterium damselae*	20.81 ± 0.19	17.46 ± 0.06
FJAT-52713	*Edwardsiella tarda*	24.25 ± 0.17	17.41 ± 0.13
FJAT-2503	*Vibrio parahaemolyticus*	16.54 ± 0.21	10.45 ± 0.15
FJAT-52716	*Vibrio vulnificus*	14.45 ± 0.13	12.54 ± 0.15
FJAT-2510	*Vibrio harveyi*	0	14.62 ± 0.25
FJAT-2512	*Vibrio alginolyticus*	0	9.93 ± 0.13

**Notes: **The anti-pathogen spectra of the strain *B. velezensis* FJAT-57093 were evaluated using the agar well diffusion assays. Ciprofloxacin and fresh LB broth were used as the positive and negative controls, respectively. Because different aquatic pathogens had varying susceptibilities to ciprofloxacin, the working concentration of ciprofloxacin to each aquatic pathogen was optimized separately. Accordingly, the concentrations of ciprofloxacin were determined to be used as follows: 0.064 µg·mL^−1^ for the strain FJAT-52715; 0.25 µg·mL^−1^ for the strains FJAT-52713, FJAT-52714, and FJAT-52716; and 1.5 µg·mL^−1^ for strains FJAT-2503, FJAT-2510, and FJAT-2512. All experiments were conducted with three independent replicates. Data were presented as the mean ± standard deviation.

## Data Availability

The original contributions presented in this study are included in the article and Supplementary Materials. Further inquiries can be directed to the corresponding authors.

## Supplementary Materials

The following supporting information can be downloaded at: https://www.mdpi.com/article/10.3390/microorganisms14010041/s1, Figure S1: Heatmap of the FJAT-57093 genome with phylogenetically related *Bacillus* strains based on the ANI values; Figure S2: Circular genomic map of the strain *B. velezensis* FJAT-57093; Figure S3: Mapping virulence genes of FJAT 57093 to pangenome categories; Table S1: The 111 *Bacillus*-like strains used to evaluate inhibitory activities against Aeromonas hydrophila in this study; Table S2: Putative virulence associated genes predicted in the genome of *B. velezensis* FJAT-57093; Table S3: Putative antibiotic resistance associated genes predicted in the genome of *B. velezensis* FJAT-57093; Table S4: Statistics of transferable elements carrying virulence and resistance genes in strain FAJT-57093; Table S5: Pangenome categories of 434 B. elezensis genomes; Table S6: Biosynthetic gene clusters of secondary metabolite predicted in the genome of *B. velezensis* FJAT-57093.
