# Supplementary material for: Probiotic Properties In Vitro of Bacillus velezensis FJAT-57093 with Antibacterial Activity Against the Aquatic Pathogen Aeromonas hydrophila
Source: Microorganisms. 2025 Dec 23;14(1):41. doi: 10.3390/microorganisms14010041 (PMC12844275; doi:10.3390/microorganisms14010041)
Supplement: Supplementary file 1 [file microorganisms-14-00041-s001.zip › microorganisms-4024550-supplementary.pdf]

Title: Probiotic properties of *Bacillus velezensis* FJAT-57093 with Antagonistic antagonistic Strains activity Against against the aquatic pathogen *Aeromonas hydrophila*

Yanping Chen[1], Suyi Li [2], Wenjie Li[1], Xuefang Zheng[1], Meichun Chen[1], Xin Liu[1], Jieping Wang[1*]

1. Institute of Resources, Environment and Soil Fertilizer, Fujian Academy of Agricultural Sciences, Fuzhou 350003, China

2. Institute of Biotechnology, Fujian Academy of Agricultural Sciences, Fuzhou, Fujian 350003, China

* **Correspondence :** wangjieping2011@163.com; lanfz2008@163.com

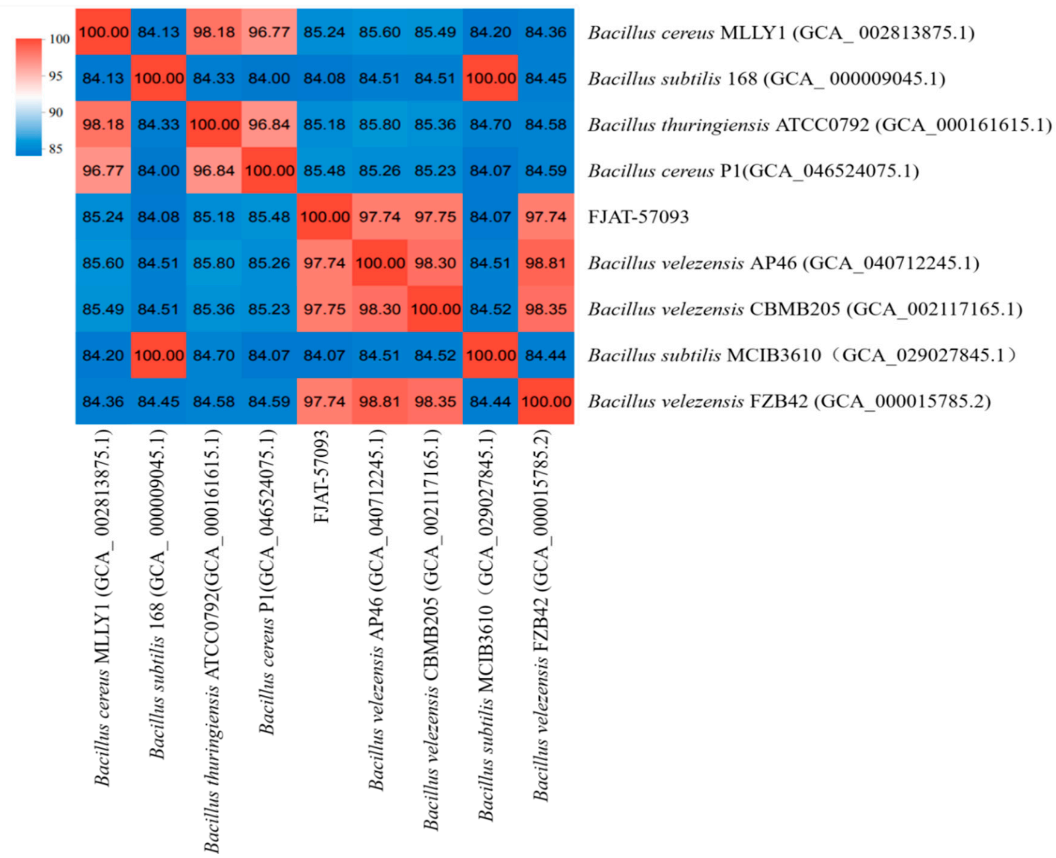

Supplementary Figure S1. Heatmap of the FJAT-57093 genome with phylogenetically related *Bacillus* strains based on the ANI values

Notes: The heatmap analysis was performed through the online tool of Majorbio Cloud Platform (https://www.majorbio.com/tools) (Han et al., 2024). The phylogenetically-related *Bacillus* strains and their genomes included *Bacillus cereus* MLLY1 (GCA 002813875.1), *B. cereus* P1 (GCA 046524075.1), *B. subtilis* 168 (GCA 000009045.1), *B. subtilis* MCIB3610 (GCA_029027845.1), *B. thuringiensis*

ATCC0792 (GCA 000161615.1), *B. velezensis* AP46 (GCA 040712245.1), *B. velezensis* CBMB205 (GCA 002117165.1) and *B. velezensis* FZB42 (GCA 000015785.2).

Chang Han, Caiping Shi, Linmeng Liu, Jichen Han, Qianqian Yang, Yan Wang, Xiaodan Li, Wenyao Fu, Hao Gao, Huasheng Huang, Xianglin Zhang, Kegang Yu. Majorbio Cloud 2024: Update single-cell and multiomics workflows. iMeta, 2024, 3(4):e217.

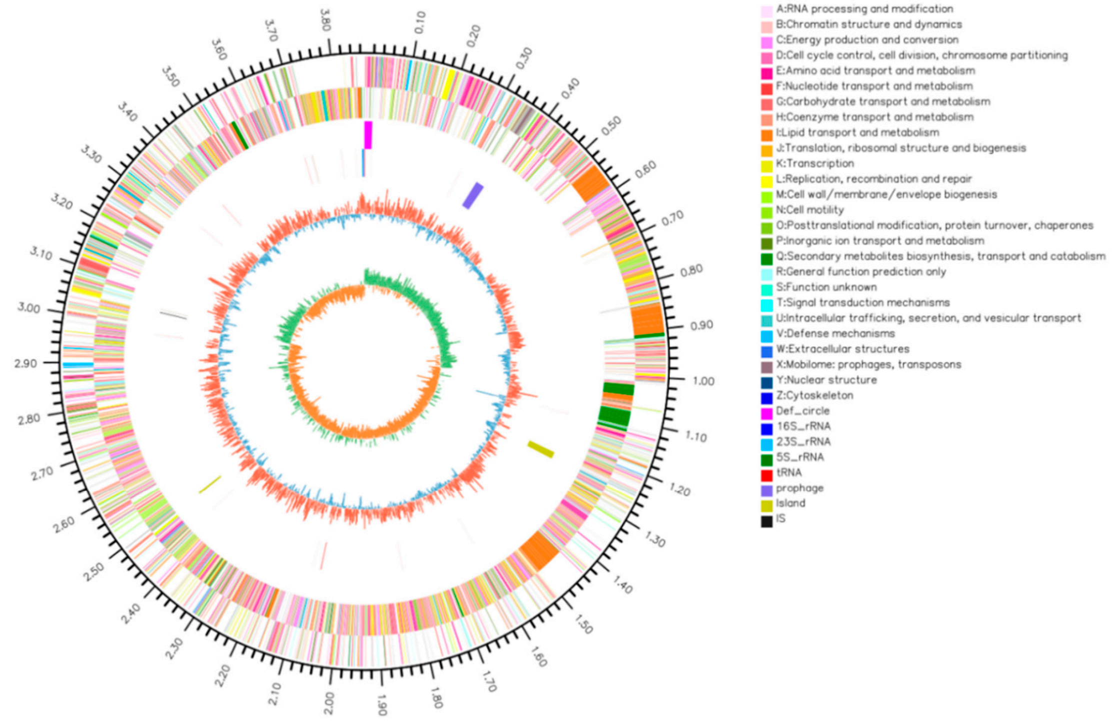

Supplementary Figure S2. Circular genomic map of the strain *B. velezensis* FJAT-57093

Notes: The genome of the strain FJAT-57093 comprised a single circular chromosome of 3,452,556 bp, with an average G+C content of 46.49% and no detectable plasmid. A total of 3,730 protein-coding genes were predicted. From outside to inside, the rings represent: Ring 1, Genome scale; Rings 2 and 3, CDS on the positive and negative strands colored by COG functional categories; Ring 4, rRNA and tRNA; Ring 5, ncRNA, prophage, genomic islands (GI), and insertion sequences (IS); Ring 6, GC content. Outward red peaks indicate regions where GC content is higher than the genomic average, with peak height reflecting the degree of deviation; inward blue peaks indicate regions where GC content is lower than the average; Ring 7: GC-skew calculated as (G-C)/(G+C), generally, positive skew (>0) corresponds to the leading strand, while negative skew (<0) corresponds to the lagging strand.

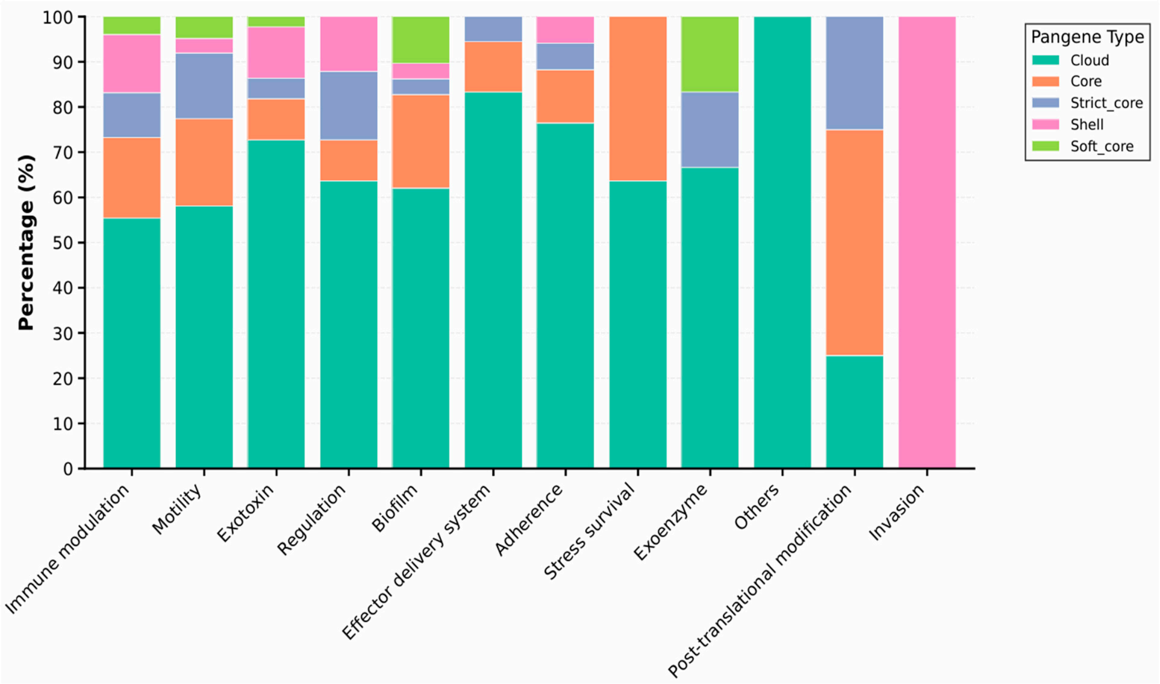

Supplementary Figure S3. Mapping virulence genes of FJAT 57093 to pangenome categories

Notes: To evaluate whether the putative virulence factors are typical, low-risk housekeeping, or high-risk features, a pangenome analysis of 434 *B. velezensis* genomes (including all NCBI complete genomes and FJAT 57093) were performed using PGAP2 (https://github.com/bucongfan/PGAP2) (Bu et al., 2025) to map virulence genes of FJAT 57093 to pangenome categories.

Bu,C.F.; Zhang,H.; Zhang,F.N.; Liang,W.H.; Gao,H.; Zhao,J.; Lv,F.M.; Xue,R.K.; Liu,Q.; Zhang,Z.W.; Jin,Z.; Xiao,J.F. PGAP2: A comprehensive toolkit for prokaryotic pan-genome analysis based on fine-grained feature networks. *Nat Commun*, **2025**, 16(1):9865.

Table S1 The 111 *Bacillus*-like strains used to evaluate inhibitory activities against *Aeromonas hydrophila* in this study

**No.**	**Strain Number**[#]	**Species Name**[*]	**Source of Isolation**	**Antibacterial Activity**
1	FJAT-520	*Bacillus coagulans*	Gift from the Culture Collection University of Gothenburg, Sweden	-
2	FJAT-2349	*Bacillus velezensis*	Rhizosphere soil of weeds, Fuzhou City, Fujian Province	+++
3	FJAT-4854	*Bacillus subtilis*	Pig feed from a pig farm in Fuqing City, Fujian Province	+
4	FJAT-4857	*Bacillus coagulans*	Pig feed from a pig farm in Fuqing City, Fujian Province	-
5	FJAT-5545	*Bacillus subtilis*	Soil from a vineyard, Putian City, Fujian Province	+
6	FJAT-7382	*Bacillus-pumilus*	Pig feed from the pig farm of Ningde Nanyang Industrial Company, Fujian Province	-
7	FJAT-8754	*Bacillus amyloliquefaciens*	Gift from the Culture Collection University of Gothenburg, Sweden	-

8	FJAT-8763	*Bacillus coagulans*	Gift from the Culture Collection University of Gothenburg, Sweden	+
9	FJAT-8779	*Bacillus pumilus*	Gift from the Culture Collection University of Gothenburg, Sweden	-
10	FJAT-8853	*Bacillus pumilus*	pig bedding from Xindian Pig Farm, Fuzhou City, Fujian Province	-
11	FJAT-10003	*Paenibacillus amylolyticus*	Gift from the Culture Collection University of Gothenburg, Sweden	-
12	FJAT-10501	*Bacillus subtilis*	pig bedding from Xindian Pig Farm, Fuzhou City, Fujian Province	+
13	FJAT-10503	*Bacillus licheniformis*	pig bedding from Xindian Pig Farm, Fuzhou City, Fujian Province	-
14	FJAT-14785	*Bacillus amyloliquefaciens*	Pig feces from a pig farm in Jian'ou City, Fujian Province	-
15	FJAT-14791	*Bacillus subtilis*	Pig feces from Yuanmuxiang Pig Farm, Fujian Province	-
16	FJAT-17931	*Bacillus velezensis*	Soil from a mango orchard, Putian City, Fujian Province	+++
17	FJAT-20278	*Bacillus pumilus*	Rhizosphere soil of needle grass by Xiao Chaidan Lake, Qinghai Province	+
18	FJAT-25658	*Bacillus amyloliquefaciens*	Paddy field, Acheng District, Harbin City	+
19	FJAT-26756	*Bacillus amyloliquefaciens*	Rhizosphere soil of soybean from the Base of Heilongjiang Academy of Agricultural Sciences	+
20	FJAT-27642	*Bacillus licheniformis*	pig bedding from a pig farm in Fuzhou City, Fujian Province	-
21	FJAT-41160	*Bacillus licheniformis*	Pig bedding from Yuxi Pig Farm, Fuqing City, Fujian Province	-
22	FJAT-41518	*Bacillus subtilis*	Pig bedding from Yuxi Pig Farm, Fuqing City, Fujian Province	+
23	FJAT-41678	*Bacillus amyloliquefaciens*	Pig bedding from Yuxi Pig Farm, Fuqing City, Fujian Province	-
24	FJAT-45100	*Bacillus pumilus*	Rhizosphere soil of weeds in saline-alkali land, Nyima County, Tibet	+
25	FJAT-45277	*Bacillus pumilus*	Rhizosphere soil of weeds in saline-alkali land, Nyima County, Tibet	-
26	FJAT-46513	*Bacillus amyloliquefacien*	Intertidal mud from a tidal flat, Ningde City, Fujian Province	++
27	FJAT-51803	*Bacillus licheniformis*	Intertidal mud from the Mangrove Reserve, Zhangzhou City, Fujian Province	-
28	FJAT-52662	*Bacillus licheniformis*	Soybean meal from Nanjing Zhirun Biological Company	-
29	FJAT-52676	*Bacillus licheniformis*	Soybean meal from Nanjing Zhirun Biological Company	-
30	FJAT-53034	*Bacillus subtilis*	Intertidal mud from Yunxiao Mangrove Wetland Park, Zhangzhou City, Fujian Province	+
31	FJAT-54144	*Bacillus subtilis*	Intertidal mud from Yunxiao Mangrove Wetland Park, Zhangzhou City, Fujian Province	+
32	FJAT-54371	*Bacillus subtilis*	Substrate soil from Yuxi Jiangping Company, Fujian Province	+
33	FJAT-55193	*Bacillus subtilis*	Endophyte of Giant Juncao grass, Nanping City, Fujian Province	-
34	FJAT-57092	*Bacillus haynesii*	Rhizosphere soil of summer squash, Gansu Province	-
35	FJAT-57093	*Bacillus velezensis*	Rhizosphere soil of summer squash, Gansu Province	++++
36	FJAT-57094	*Bacillus proteolyticus*	Rhizosphere soil of summer squash, Gansu Province	-
37	FJAT-57095	*Peribacillus simplex*	Rhizosphere soil of summer squash, Gansu Province	-
38	FJAT-57096	*Priestia megaterium*	Rhizosphere soil of summer squash, Gansu Province	-
39	FJAT-57097	*Rossellomorea arthrocnemi*	Rhizosphere soil of summer squash, Gansu Province	-
40	FJAT-57098	*Cytobacillus firmus*	Rhizosphere soil of summer squash, Gansu Province	-
41	FJAT-57099	*Peribacillus frigoritolerans*	Rhizosphere soil of summer squash, Gansu Province	-
42	FJAT-57100	*Bacillus zhangzhouensis*	Rhizosphere soil of weeds, Gansu Province	-
43	FJAT-57101	*Bacillus zhangzhouensis*	Rhizosphere soil of weeds, Gansu Province	+
44	FJAT-57102	*Bacillus swezeyi*	Rhizosphere soil of weeds, Gansu Province	++
45	FJAT-57103	*Peribacillus frigoritolerans*	Rhizosphere soil of silage corn, Gansu Province	-
46	FJAT-57104	*Bacillus rugosus*	Rhizosphere soil of silage corn, Gansu Province	++
47	FJAT-57105	*Priestia megaterium*	Rhizosphere soil of silage corn, Gansu Province	-
48	FJAT-57106	*Priestia megaterium*	Rhizosphere soil of silage corn, Gansu Province	-
49	FJAT-57107	*Bacillus haynesii*	Rhizosphere soil of silage corn, Gansu Province	-
50	FJAT-57108	*Bacillus thuringiensis*	Rhizosphere soil of silage corn, Gansu Province	+++
51	FJAT-57109	*Priestia megaterium*	Rhizosphere soil of silage corn, Gansu Province	-
52	FJAT-57110	*Bacillus mobilis*	Rhizosphere soil of silage corn, Gansu Province	++
53	FJAT-57111	*Priestia megaterium*	Rhizosphere soil of fennel, Gansu Province	-
54	FJAT-57112	*Bacillus australimaris*	Rhizosphere soil of fennel, Gansu Province	-
55	FJAT-57114	*Priestia megaterium*	Rhizosphere soil of fennel, Gansu Province	+
56	FJAT-57115	*Priestia flexa*	Rhizosphere soil of fennel, Gansu Province	+
57	FJAT-57116	*Priestia megaterium*	Rhizosphere soil of fennel, Gansu Province	-
58	FJAT-57117	*Bacillus velezensis*	Rhizosphere soil of fennel, Gansu Province	+++
59	FJAT-57118	*Bacillus thuringiensis*	Rhizosphere soil of fennel, Gansu Province	++
60	FJAT-57119	*Bacillus australimaris*	Rhizosphere soil of alfalfa, Gansu Province	-
61	FJAT-57120	*Priestia megaterium*	Rhizosphere soil of alfalfa, Gansu Province	-
62	FJAT-57121	*Bacillus atrophaeus*	Rhizosphere soil of alfalfa, Gansu Province	-
63	FJAT-57122	*Peribacillus frigoritolerans*	Rhizosphere soil of alfalfa, Gansu Province	-
64	FJAT-57123	*Bacillus zhangzhouensis*	Rhizosphere soil of alfalfa, Gansu Province	-
65	FJAT-57124	*Bacillus proteolyticus*	Rhizosphere soil of alfalfa, Gansu Province	-

66	FJAT-57125	*Bacillus proteolyticus*	Rhizosphere soil of alfalfa, Gansu Province	-
67	FJAT-57126	*Bacillus proteolyticus*	Rhizosphere soil of alfalfa, Gansu Province	-
68	FJAT-57407	*Peribacillus frigoritolerans*	Soil from a pear orchard, Gansu Province	-
69	FJAT-57408	*Bacillus atrophaeus*	Soil from a pear orchard, Gansu Province	-
70	FJAT-57409	*Bacillus mycoides*	Soil from a pear orchard, Gansu Province	-
71	FJAT-57410	*Paenibacillus lautus*	Soil from a pear orchard, Gansu Province	-
72	FJAT-57411	*Bacillus aerius*	Soil from a pear orchard, Gansu Province	-
73	FJAT-57412	*Bacillus wiedmannii*	Soil from a pear orchard, Gansu Province	-
74	FJAT-57413	*Peribacillus frigoritolerans*	Soil from a pear orchard, Gansu Province	-
75	FJAT-57414	*Psychrobacillus psychrodurans*	Soil from a pear orchard, Gansu Province	+
76	FJAT-57415	*Bacillus proteolyticus*	Rhizosphere soil of Welsh onion, Gansu Province	+
77	FJAT-57417	*Bacillus pumilus*	Rhizosphere soil of Welsh onion, Gansu Province	++
78	FJAT-57418	*Bacillus mycoides*	Rhizosphere soil of Welsh onion, Gansu Province	+
79	FJAT-57419	*Bacillus atrophaeus*	Rhizosphere soil of Welsh onion, Gansu Province	+++
80	FJAT-57420	*Peribacillus frigoritolerans*	Rhizosphere soil of Welsh onion, Gansu Province	-
81	FJAT-57421	*Mesobacillus thioparans*	Rhizosphere soil of Welsh onion, Gansu Province	-
82	FJAT-57422	*Priestia megaterium*	Rhizosphere soil of Welsh onion, Gansu Province	-
83	FJAT-57423	*Bacillus mycoides*	Rhizosphere soil of Welsh onion, Gansu Province	-
84	FJAT-57424	*Paenibacillus tundrae*	Rhizosphere soil of Welsh onion, Gansu Province	-
85	FJAT-57425	*Priestia megaterium*	Rhizosphere soil of lambsquarters, Gansu Province	-
86	FJAT-57426	*Rossellomorea marisflavi*	Rhizosphere soil of lambsquarters, Gansu Province	-
87	FJAT-57427	*Peribacillus frigoritolerans*	Rhizosphere soil of lambsquarters, Gansu Province	-
88	FJAT-57428	*Paenibacillus lautus*	Rhizosphere soil of lambsquarters, Gansu Province	-
89	FJAT-57429	*Bacillus thuringiensis*	Rhizosphere soil of lambsquarters, Gansu Province	-
90	FJAT-57430	*Peribacillus frigoritolerans*	Rhizosphere soil of lambsquarters, Gansu Province	-
91	FJAT-57431	*Bacillus mojavensis*	Rhizosphere soil of lambsquarters, Gansu Province	-
92	FJAT-57432	*Peribacillus frigoritolera*	Rhizosphere soil of lambsquarters, Gansu Province	-
93	FJAT-57433	*Peribacillus frigoritolerans*	Rhizosphere soil of lambsquarters, Gansu Province	-
94	FJAT-57434	*Peribacillus frigoritolerans*	Rhizosphere soil of lambsquarters, Gansu Province	-
95	FJAT-57435	*Bacillus proteolyticus*	Rhizosphere soil of corn, Gansu Province	-
96	FJAT-57436	*Peribacillus frigoritolerans*	Rhizosphere soil of corn, Gansu Province	-
97	FJAT-57437	*Rossellomorea marisflavi*	Rhizosphere soil of corn, Gansu Province	-
98	FJAT-57438	*Peribacillus frigoritolerans*	Rhizosphere soil of corn, Gansu Province	-
99	FJAT-57439	*Bacillus velezensis*	Rhizosphere soil of corn, Gansu Province	++
100	FJAT-57440	*Bacillus haynesii*	Rhizosphere soil of corn, Gansu Province	-
101	FJAT-57441	*Peribacillus simplex*	Rhizosphere soil of corn, Gansu Province	-
102	FJAT-57442	*Peribacillus frigoritolerans*	Rhizosphere soil of corn, Gansu Province	-
103	FJAT-57443	*Priestia megaterium*	Soil from a pear orchard, Gansu Province	-
104	FJAT-57444	*Bacillus pumilus*	Soil from a pear orchard, Gansu Province	+++
105	FJAT-57445	*Peribacillus simplex*	Soil from a pear orchard, Gansu Province	-
106	FJAT-57446	*Bacillus atrophaeus*	Soil from a pear orchard, Gansu Province	++
107	FJAT-57447	*Bacillus wiedmannii*	Soil from a pear orchard, Gansu Province	-
108	FJAT-57448	*Paenibacillus dongdonensis*	Soil from a pear orchard, Gansu Province	-
109	FJAT-57449	*Oikeobacillus pervagus*	Soil from a pear orchard, Gansu Province	-
110	FJAT-57450	*Peribacillus frigoritolerans*	Soil from a pear orchard, Gansu Province	-
111	FJAT-57678	*Bacillus subtilis*	Chicken manure from Shennong Chicken Farm, Fujian Province	+

Note: # The strain number (FJAT-) was authorized by the Fujian Bacilli Resource Collection Center (FBRCC), Institute of Resources, Environment and Soil Fertilizer, Fujian Academy of Agricultural Sciences. * The taxonomic information of each strain had been validated through 16S rRNA and *gyrB* sequence analyses by FBRCC. "+", "++", "+++", and "++++" indicated that the antibacterial diameter is between 5 and 10 mm, 10

and 15 mm, 15 and 20 mm, and more than 20 mm, respectively. "-" indicated that the antibacterial activity was not detected. All the antibacterial screening assays were performed in triplicate.

Table S2. Putative virulence associated genes predicted in the genome of *B. velezensis* FJAT-57093.

NO	Functioin description	Gene name	Genes number
1	Adherence	*cgsD, cwp66, ebpB, fbpA, fimZ, gbpA, groEL, lap, lmb, pebA, pilD, pilJ, pilS, rpoS, scpA, scpB, tufA*	16
2	Biofilm	*algC, algI, algR, algW, algZ, bopD, icaA, icaC, icaR, mucD, mucP, pgaC, vpsC, vpsG, vpsI, vpsU*	16
3	Effector delivery system	*CBU_1566, CT_061, LPG_RS11860, bapC, bprB, cdsN, dotU1, eccA3, espK, essC, glgX, ppkA, pppA, ricA, ssrA, ssrB, vasH, xcpR, yycJ*	19
4	Exoenzyme	*aur, cwp84, tlyC*	3
5	Exotoxin	*cesA, cesB, cesC, cesP, clbB, clbD, clbF, clbG, cyaB, cylA, cylB, cylG, hlyB, plcD, rtxB*	15
6	Immune modulation	*ACICU_RS00475, ACICU_RS00485, Cj1135, FTT_RS04105, FTT_RS04145, GBS_RS06565, GBS_RS06600, LPG_RS03740, LPG_RS03745, YE_RS15445, acpXL, adsA, bplF, bplI, cap8B, cap8C, cap8D, cap8E, cap8O, capA, capB, capC, cps4H, cps4I, cpsA/uppS, cpsB/cdsA, cpsC, cpsG, cpsJ, cpsK, cysC, ddrA, fabZ, flmF2, galE, gndA, gtrB, hasC, kdtB, kfiC, legI, lgtF, lpxA/glmU, lsgE, manB/yhxB, mas, mmpL8, msbA, oatA, orfM, pdgA, per, pks1, pks2, ppsA, ppsB, ppsC, rfaE, rfbA, rfbD, rfbF, rpe, wbpA, wbpL, wbtH, wbtM, wbuZ, wcbT, wlaN*	69
7	Invasion	*aut, iap, cwhA*	2
8	Motility	*AHML_RS07540, LPG_RS02380, LPG_RS13035, PA3348, cheA, cheB, cheB-2, cheV, cheV3, cheW, flaA, fleN, fleQ, fleR/flrC, fleS, fleS/flrB, flgB, flgC, flgD, flgG2, flgG_2, flhA, flhB, flhF, flhG, fliA, fliD, fliE, fliF, fliG, fliI, fliM, fliN, fliP, fliQ, fliR, fliS, flmH, lafK, lfgK, lfgL, motA, motD, tlpA, tlpB*	45
9	Others	*aatC, acfB*	2
10	Post-translational modification	*gtcA, lspA, prsA2*	3
11	Regulation	*AAD32423, bvgA, bvgS, bvrR, csrA, devR/dosR, devS, fur, mprA, phoP, phoQ, phoR, rcsB, relA, sigA/rpoV, sigE, sigF*	17
12	Stress survival	*clpC, clpE, clpP, katA, msrA/BpilB, recN, sodA, sodB, ureA, ureB*	10

Notes: The putative virulence associated genes were predicted in the genome of *B. velezensis* FJAT-57093 using the Virulence Factor Database (VFDB) (Liu et al., 2022).

Liu B, Zheng D, Zhou S, Chen L, Yang J. VFDB 2022: a general classification scheme for bacterial virulence factors. Nucleic Acids Res, 2022, 50(D1):D912-D917.

Table S3. Putative antibiotic resistance associated genes predicted in the genome of *B. velezensis* FJAT-57093.

No	Functioin description	Gene name	Genes number
1	aminocoumarin antibiotic	*muxC, gyrB, abeS, baeR, novA*	5
2	aminoglycoside antibiotic	*rpsL, tlyA, ranA, lmrS, aadK, baeR, kdpD, smeR, ykkC, ykkD*	10
3	antibacterial free fatty acids	*mtrR*	1
4	bicyclomycin-like antibiotic	*bcr-1*	1
5	carbapenem	*CAR-1, soxS, pbp1, PBP2, PEDO-1, PBP1a, PBP2b, PBP2x, golS, mecA*	10
6	cephalosporin	*bcII, acrR, soxS, pbp1, PBP2, SRT-1, SST-1, PBP1a, PBP2b, PBP2x, golS, mecA, mgrA, smeR*	14
7	cephamycin	*soxS, pbp1, PBP2, PBP1a, PBP2b, PBP2x, golS, mecA, smeR*	9
8	diaminopyrimidine antibiotic	*LmrS, dfrG, rsmA*	3
9	disinfecting agents and antiseptics	*acrR, fabG, fabI, soxS, kasA, abeM, arlR, arlS, blt, mgrA, qacE, qacL, sdrM*	13
10	elfamycin antibiotic	*EF-Tu, facT*	2
11	fluoroquinolone antibiotic	*acrR, soxS, gyrA, norA, parC, parE, abeM, arlR, arlS, blt, efmA, efrA, emrB, evgA, evgS, mgrA, patA, patB, pmrA, qacA, rsmA, sdrM*	22
12	fusidane antibiotic	*fusA, fusE*	2
13	glycopeptide antibiotic	*murG, D-Ala-D-Ala, rpld, vanH, vanR, vanS, vanT, vanXY*	8
14	glycylcycline	*acrR, soxS, adeR*	3
15	isoniazid-like antibiotic	*fabI, kasA, mshA, ndh*	4
16	lincosamide antibiotic	*ermQ, cfrA, clbA, lmrB, lmrD, lsaC, vgaC, vmlR*	8
17	macrolide antibiotic	*ermQ, MuxC, rpld, LmrS, abeS, efmA, efrA, evgA, evgS, macB, mphK, mreA, mtrR, oleD, oleI*	15
18	monobactam	*soxS , pbp1, MuxC, PBP2, PBP1a, PBP2b, PBP2x, golS, mecA*	9
19	mupirocin-like antibiotic	*ileS, mupA*	2
20	nitrofuran antibiotic	*nfsA*	1
21	nitroimidazole antibiotic	*frxA, msbA*	2
22	nucleoside antibiotic	*SAT-4, tmrB*	2
23	nybomycin-like antibiotic	*gyrA*	1
24	oxazolidinone antibiotic	*lmrS, cfrA, clbA, mlaF, optrA*	5
25	penam	*bcII, Bla1,tetA, acrR, soxS, pbp1, PBP2, PBP1a, PBP2b, PBP2x , evgA, evgS, golS, mecA, mgrA, mtrR, smeR*	17
26	penem	*soxS, golS*	2
27	peptide antibiotic	*mprF, rpoB, yybT, liaR, liaS, liaR, liaS, PmrF, cls, menA, pgsA, rpoC, walK, cdsA, yojI, bacA, bcrA, cprR, mgrA, rosA, rosB, ugd*	22
28	phenicol antibiotic	*acrR, soxS, LmrS, cfrA, clbA, cmlv, cmx, fexA, golS, optrA, rsmA, ykkC, ykkD*	13
29	phosphonic acid antibiotic	*abaF, ptsI, fosA7.5, fosM2, glpT, murA*	6
30	pleuromutilin antibiotic	*TaeA, cfrA, clbA, lsaC, vgaC, vgaD*	6

31	pyrazine antibiotic	*rpsA*	1
32	rifamycin antibiotic	*rpoB, acrR, soxS , helR, rox, efrA, rphA, rphB*	8
33	salicylic acid antibiotic	*thyA*	1
34	streptogramin A antibiotic	*ermQ, cfrA, clbA, vatF, vgaC, vgaD*	6
35	streptogramin B antibiotic	*ErmQ, vmlR*	2
36	streptogramin antibiotic	*ermQ, cfrA, clbA, lsaC, vatF, vgaC, vgaD, vmlR*	8
37	sulfonamide antibiotic	*sul4*	1
38	tetracycline antibiotic	*tetA, acrR, soxS, muxC, txR, adeR, evgA, evgS, mgrA, rpsJ, tet(39), tet(C), tet(L), tet(T), tetA(58), tetA(60), tetB(60), ykkC, ykkD*	19

Notes: The putative antibiotic-resistance genes were predicted in the genome of *B. velezensis* FJAT-57093 using the Comprehensive Antibiotic Resistance Database (CARD) (Alcock et al., 2023).

Alcock BP, Huynh W, Chalil R, Smith KW, Raphenya AR, Wlodarski MA, Edalatmand A, Petkau A, Syed SA, Tsang KK, Baker SJC, Dave M, McCarthy MC, Mukiri KM, Nasir JA, Golbon B, Imtiaz H, Jiang X, Kaur K, Kwong M, Liang ZC, Niu KC, Shan P, Yang JYJ, Gray KL, Hoad GR, Jia B, Bhando T, Carfrae LA, Farha MA, French S, Gordzevich R, Rachwalski K, Tu MM, Bordeleau E, Dooley D, Griffiths E, Zubyk HL, Brown ED, Maguire F, Beiko RG, Hsiao WWL, Brinkman FSL, Van Domselaar G, McArthur AG. CARD 2023: expanded curation, support for machine learning, and resistome prediction at the Comprehensive Antibiotic Resistance Database. Nucleic Acids Res, 2023, 51(D1):D690-D699.

Table S4. Statistics of transferable elements carrying virulence and resistance genes in strain FAJT-57093

contig	geneID	strat	end	ARG	VF	mobile genetic elements
Scaffold1	gene0333	299672	300586	*Bla1*	/	Scaffold1:compositional_outlier(292140:300345)
Scaffold1	gene0720	720013	720753		*flmH*	Scaffold1:compositional_outlier(719782:727992)
Scaffold1	gene0783	777516	778304	*Streptococcus mitis CdsA with mutation conferring daptomycin resistance*	*cpsB/cdsA*	Scaffold1:compositional_outlier(772223:777715)
Scaffold1	gene1138	1237930	1238355	/	*cap8E*	Scaffold1:compositional_outlier(1237305:1257538)
Scaffold1	gene1162	1256615	1257187	/	*cap8D*	Scaffold1:compositional_outlier(1237305:1257538)
Scaffold1	gene1163	1257228	1257662	/	*msrA/B pilB*	Scaffold1:compositional_outlier(1237305:1257538)
Scaffold1	gene1384	1461351	1476965	/	*ppsB*	Scaffold1:compositional_outlier(1464626:1470221)
Scaffold1	gene1387	1489086	1501667	/	*ppsA*	Scaffold1:compositional_outlier(1489586:1495389)
Scaffold2	gene2015	22346	23176	*bacA*	/	Scaffold2:compositional_outlier(12439:22634)

Scaffold2	gene2069	75873	76517	*Enterococcus faecium liaR mutant conferring daptomycin resistance*	*ssrB*	Scaffold2:compositional_outlier(74680:82948)
Scaffold2	gene2070	76598	77194	*Enterococcus faecium liaS mutant conferring daptomycin resistance*	/	Scaffold2:compositional_outlier(74680:82948)
Scaffold2	gene2090	92309	92524	/	*mbtH-like*	Scaffold2:compositional_outlier(92018:97972)
Scaffold2	gene2091	92543	99670	/	*dhbF*	Scaffold2:compositional_outlier(92018:97972)
Scaffold3	gene3102	111688	112578	*tetA(58)*	*ddrA*	Scaffold3:compositional_outlier(109614:115410)
Scaffold4	gene3330	82740	83177	*mgrA*	/	Scaffold4:compositional_outlier(74668:90467)
Scaffold4	gene3333	85603	87006	*Acinetobacter baumannii AbaF*	/	Scaffold4:compositional_outlier(74668:90467)

Table S5. Pangenome categories of 434 *B. elezensis* genomes

Pangenome categories	Criteria	Gene count	Percentage
Strict core genes	(strains = 100%)	411	1.48%
Core genes	(99% <= strains < 100%)	1627	5.86%
Soft core genes	(95% <= strains < 99%)	945	3.40%
Shell genes	(15% <= strains < 95%)	1462	5.26%
Cloud genes	(0% <= strains < 15%)	23339	84.00%
Total genes	(0% <= strains <= 100%)	27784	100.00%

Notes: The pangenome analysis of 434 *B. velezensis* genomes (including all NCBI complete genomes and FJAT 57093) was performed using PGAP2 (https://github.com/bucongfan/PGAP2) (Bu et al., 2025).

Table S6. Biosynthetic gene clusters of secondary metabolite predicted in the genome of *B. velezensis* FJAT-57093.

Gene ID	Location	Cluster ID	NR Description	Function
gene0084	Scaffold1	cluster1	MULTISPECIES: nitric oxide-sensing transcriptional repressor NsrR	other
gene0085	Scaffold1	cluster1	hypothetical protein	other
gene0086	Scaffold1	cluster1	MULTISPECIES: SpoVR family protein	other
gene0087	Scaffold1	cluster1	MULTISPECIES: alkaline phosphatase	other
gene0088	Scaffold1	cluster1	MULTISPECIES: C40 family peptidase	other
gene0089	Scaffold1	cluster1	MULTISPECIES: LysR family transcriptional regulator	regulatory
gene0090	Scaffold1	cluster1	citrate synthase I	other
gene0091	Scaffold1	cluster1	MULTISPECIES: SDR family oxidoreductase	biosynthetic-add

				itional
gene0092	Scaffold1	cluster1	MULTISPECIES: amino acid permease	biosynthetic-additional
gene0093	Scaffold1	cluster1	sodium-dependent transporter	other
gene0094	Scaffold1	cluster1	MULTISPECIES: anti-sigma-M factor	other
gene0095	Scaffold1	cluster1	putative anti-sigma-M factor yhdL	other
gene0096	Scaffold1	cluster1	MULTISPECIES: RNA polymerase sigma factor SigM	regulatory
gene0097	Scaffold1	cluster1	MULTISPECIES: 1-acylglycerol-3-phosphate O-acyltransferase	biosynthetic-additional
gene0098	Scaffold1	cluster1	MULTISPECIES: hemolysin family protein	other
gene0099	Scaffold1	cluster1	MULTISPECIES: MerR family transcriptional regulator	regulatory
gene0100	Scaffold1	cluster1	MULTISPECIES: pyridoxal phosphate-dependent aminotransferase	biosynthetic-additional
gene0101	Scaffold1	cluster1	MULTISPECIES: beta-ketoacyl synthase N-terminal-like domain-containing protein	biosynthetic
gene0102	Scaffold1	cluster1	MULTISPECIES: hemolysin family protein	other
gene0103	Scaffold1	cluster1	MULTISPECIES: fluoride efflux transporter CrcB	other
gene0104	Scaffold1	cluster1	MULTISPECIES: CrcB family protein	other
gene0105	Scaffold1	cluster1	MULTISPECIES: glycerophosphodiester phosphodiesterase family protein	other
gene0106	Scaffold1	cluster1	MULTISPECIES: YhdX family protein	other
gene0107	Scaffold1	cluster1	MULTISPECIES: mechanosensitive ion channel family protein	other
gene0108	Scaffold1	cluster1	MULTISPECIES: NAD-dependent protein deacylase	other
gene0109	Scaffold1	cluster1	MULTISPECIES: polysaccharide deacetylase family protein	biosynthetic-additional
gene0110	Scaffold1	cluster1	MULTISPECIES: D-amino-acid transaminase	other
gene0111	Scaffold1	cluster1	transcriptional regulator	regulatory
gene0112	Scaffold1	cluster1	MULTISPECIES: hypothetical protein	other
gene0113	Scaffold1	cluster1	MULTISPECIES: universal stress protein	other
gene0114	Scaffold1	cluster1	hypothetical protein	other
gene0115	Scaffold1	cluster1	MULTISPECIES: ABC transporter transmembrane domain-containing protein	transport
gene0116	Scaffold1	cluster1	MULTISPECIES: ABC transporter ATP-binding protein	transport
gene0117	Scaffold1	cluster1	MULTISPECIES: SDR family oxidoreductase	other
gene0118	Scaffold1	cluster1	MULTISPECIES: hypothetical protein	other
gene0119	Scaffold1	cluster1	MULTISPECIES: alpha/beta-type small acid-soluble spore protein	other
gene0120	Scaffold1	cluster1	MULTISPECIES: YheE family protein	other
gene0121	Scaffold1	cluster1	MULTISPECIES: YheC/YheD family protein	other
gene0122	Scaffold1	cluster1	MULTISPECIES: YheC/YheD family protein	other
gene0123	Scaffold1	cluster1	MULTISPECIES: DUF445 family protein	other
gene0124	Scaffold1	cluster1	MULTISPECIES: YlbF family regulator	other
gene0204	Scaffold1	cluster2	MULTISPECIES: hypothetical protein	other
gene0205	Scaffold1	cluster2	MULTISPECIES: spore germination protein	other
gene0206	Scaffold1	cluster2	MULTISPECIES: spore germination protein GerPE	other
gene0207	Scaffold1	cluster2	MULTISPECIES: hypothetical protein	other
gene0208	Scaffold1	cluster2	spore germination protein GerPC	other
gene0209	Scaffold1	cluster2	MULTISPECIES: spore germination protein GerPB	other
gene0210	Scaffold1	cluster2	MULTISPECIES: spore germination protein	other
gene0211	Scaffold1	cluster2	MULTISPECIES: aspartyl-phosphate phosphatase Spo0E family	other

			protein	
gene0212	Scaffold1	cluster2	MULTISPECIES: fumarylacetoacetate hydrolase family protein	other
gene0213	Scaffold1	cluster2	MULTISPECIES: YisL family protein	other
gene0214	Scaffold1	cluster2	MULTISPECIES: DUF2777 family protein	other
gene0215	Scaffold1	cluster2	MULTISPECIES: asparagine synthase (glutamine-hydrolyzing)	biosynthetic-additional
gene0216	Scaffold1	cluster2	MULTISPECIES: squalene/phytoene synthase family protein	biosynthetic
gene0217	Scaffold1	cluster2	hypothetical protein	transport
gene0218	Scaffold1	cluster2	MULTISPECIES: AraC family transcriptional regulator	regulatory
gene0219	Scaffold1	cluster2	MULTISPECIES: LacI family DNA-binding transcriptional regulator	regulatory
gene0220	Scaffold1	cluster2	MULTISPECIES: inositol 2-dehydrogenase	biosynthetic-additional
gene0221	Scaffold1	cluster2	MULTISPECIES: DinB family protein	other
gene0222	Scaffold1	cluster2	MULTISPECIES: LysE/ArgO family amino acid transporter	transport
gene0223	Scaffold1	cluster2	MULTISPECIES: PLP-dependent aminotransferase family protein	biosynthetic-additional
gene0224	Scaffold1	cluster2	MULTISPECIES: pentapeptide repeat-containing protein	other
gene0225	Scaffold1	cluster2	MULTISPECIES: GNAT family N-acetyltransferase	other
gene0563	Scaffold1	cluster3	MULTISPECIES: 1-phosphofructokinase	biosynthetic-additional
gene0564	Scaffold1	cluster3	MULTISPECIES: PTS fructose transporter subunit IIABC	other
gene0565	Scaffold1	cluster3	MULTISPECIES: signal peptidase I	other
gene0566	Scaffold1	cluster3	MULTISPECIES: hypothetical protein	other
gene0567	Scaffold1	cluster3	MULTISPECIES: ATP-binding cassette domain-containing protein	transport
gene0568	Scaffold1	cluster3	MULTISPECIES: ketopantoate reductase family protein	other
gene0569	Scaffold1	cluster3	MULTISPECIES: aminopeptidase	other
gene0570	Scaffold1	cluster3	MULTISPECIES: protein YkpC	other
gene0571	Scaffold1	cluster3	cell-shape determining protein	other
gene0572	Scaffold1	cluster3	MULTISPECIES: AbrB/MazE/SpoVT family DNA-binding domain-containing protein	other
gene0573	Scaffold1	cluster3	MULTISPECIES: ATP-binding protein	regulatory
gene0574	Scaffold1	cluster3	MULTISPECIES: gamma-glutamylcyclotransferase family protein	other
gene0575	Scaffold1	cluster3	MULTISPECIES: Ktr system potassium transporter KtrC	other
gene0576	Scaffold1	cluster3	MULTISPECIES: adenine deaminase	other
gene0577	Scaffold1	cluster3	MULTISPECIES: ribonuclease J1	other
gene0578	Scaffold1	cluster3	MULTISPECIES: DNA-dependent RNA polymerase subunit epsilon	other
gene0579	Scaffold1	cluster3	MULTISPECIES: Cof-type HAD-IIB family hydrolase	biosynthetic-additional
gene0580	Scaffold1	cluster3	MULTISPECIES: peptide deformylase	biosynthetic-additional
gene0581	Scaffold1	cluster3	MULTISPECIES: YjcZ family sporulation protein	other
gene0582	Scaffold1	cluster3	putative protein ykyA	other
gene0583	Scaffold1	cluster3	MULTISPECIES: ACP S-malonyltransferase	biosynthetic
gene0584	Scaffold1	cluster3	MULTISPECIES: SDR family NAD(P)-dependent oxidoreductase	biosynthetic
gene0585	Scaffold1	cluster3	type I polyketide synthase	biosynthetic
gene0586	Scaffold1	cluster3	type I polyketide synthase	biosynthetic

gene0587	Scaffold1	cluster3	SDR family NAD(P)-dependent oxidoreductase	biosynthetic
gene0588	Scaffold1	cluster3	polyketide synthase	biosynthetic
gene0589	Scaffold1	cluster3	MULTISPECIES: SDR family NAD(P)-dependent oxidoreductase	biosynthetic
gene0590	Scaffold1	cluster3	MULTISPECIES: alpha/beta fold hydrolase	biosynthetic-additional
gene0591	Scaffold1	cluster3	MULTISPECIES: serine hydrolase domain-containing protein	biosynthetic-additional
gene0592	Scaffold1	cluster3	MULTISPECIES: pyruvate dehydrogenase (acetyl-transferring) E1 component subunit alpha	biosynthetic-additional
gene0593	Scaffold1	cluster3	MULTISPECIES: pyruvate dehydrogenase complex E1 component subunit beta	biosynthetic-additional
gene0594	Scaffold1	cluster3	MULTISPECIES: pyruvate dehydrogenase complex dihydrolipoyllysine-residue acetyltransferase	other
gene0595	Scaffold1	cluster3	MULTISPECIES: dihydrolipoyl dehydrogenase	biosynthetic-additional
gene0596	Scaffold1	cluster3	MULTISPECIES: hypothetical protein	other
gene0597	Scaffold1	cluster3	MULTISPECIES: polysaccharide deacetylase family protein	biosynthetic-additional
gene0598	Scaffold1	cluster3	MULTISPECIES: aminotransferase class I/II-fold pyridoxal phosphate-dependent enzyme	biosynthetic-additional
gene0599	Scaffold1	cluster3	MULTISPECIES: UPF0223 family protein	other
gene0600	Scaffold1	cluster3	MULTISPECIES: DUF1054 domain-containing protein	other
gene0601	Scaffold1	cluster3	hypothetical protein	other
gene0602	Scaffold1	cluster3	MULTISPECIES: inositol-1-monophosphatase	biosynthetic-additional
gene0603	Scaffold1	cluster3	MULTISPECIES: GNAT family N-acetyltransferase	other
gene0604	Scaffold1	cluster3	MULTISPECIES: M4 family metallopeptidase	other
gene0605	Scaffold1	cluster3	MULTISPECIES: zf-HC2 domain-containing protein	other
gene0606	Scaffold1	cluster3	MULTISPECIES: hypothetical protein	other
gene0825	Scaffold1	cluster4	MULTISPECIES: ribonuclease Y	other
gene0826	Scaffold1	cluster4	MULTISPECIES: TIGR00282 family metallophosphoesterase	other
gene0827	Scaffold1	cluster4	MULTISPECIES: stage V sporulation protein SpoVS	other
gene0828	Scaffold1	cluster4	MULTISPECIES: L-threonine 3-dehydrogenase	biosynthetic-additional
gene0829	Scaffold1	cluster4	8-amino-7-oxononanoate synthase	biosynthetic-additional
gene0830	Scaffold1	cluster4	MULTISPECIES: tRNA (N6-isopentenyl adenosine(37)-C2)-methylthiotransferase MiaB	biosynthetic-additional
gene0831	Scaffold1	cluster4	MULTISPECIES: RicAFT regulatory complex protein RicA family protein	other
gene0832	Scaffold1	cluster4	MULTISPECIES: outer spore coat protein CotE	other
gene0833	Scaffold1	cluster4	DNA mismatch repair protein MutS	other
gene0834	Scaffold1	cluster4	MULTISPECIES: DNA mismatch repair endonuclease MutL	other
gene0835	Scaffold1	cluster4	hypothetical protein	other
gene0836	Scaffold1	cluster4	MULTISPECIES: AIPR family protein	other
gene0837	Scaffold1	cluster4	MULTISPECIES: hypothetical protein	other
gene0838	Scaffold1	cluster4	MULTISPECIES: hypothetical protein	other
gene0839	Scaffold1	cluster4	putative polyketide biosynthesis zinc-dependent hydrolase BaeB	biosynthetic-additional
gene0840	Scaffold1	cluster4	MULTISPECIES: ACP S-malonyltransferase	biosynthetic

gene0841	Scaffold1	cluster4	MULTISPECIES: acyltransferase domain-containing protein	biosynthetic
gene0842	Scaffold1	cluster4	MULTISPECIES: ACP S-malonyltransferase	biosynthetic
gene0843	Scaffold1	cluster4	MULTISPECIES: acyl carrier protein	biosynthetic-additional
gene0844	Scaffold1	cluster4	MULTISPECIES: hydroxymethylglutaryl-CoA synthase family protein	biosynthetic
gene0845	Scaffold1	cluster4	MULTISPECIES: enoyl-CoA hydratase/isomerase	biosynthetic-additional
gene0846	Scaffold1	cluster4	MULTISPECIES: polyketide synthase	biosynthetic-additional
gene0847	Scaffold1	cluster4	MULTISPECIES: non-ribosomal peptide synthetase	biosynthetic
gene0848	Scaffold1	cluster4	SDR family NAD(P)-dependent oxidoreductase	biosynthetic
gene0849	Scaffold1	cluster4	MULTISPECIES: SDR family NAD(P)-dependent oxidoreductase	biosynthetic
gene0850	Scaffold1	cluster4	MULTISPECIES: non-ribosomal peptide synthetase	biosynthetic
gene0851	Scaffold1	cluster4	beta-ketoacyl synthase N-terminal-like domain-containing protein	biosynthetic
gene0852	Scaffold1	cluster4	MULTISPECIES: cytochrome P450	biosynthetic-additional
gene0853	Scaffold1	cluster4	MULTISPECIES: NucA/NucB deoxyribonuclease domain-containing protein	other
gene0854	Scaffold1	cluster4	hypothetical protein	other
gene0855	Scaffold1	cluster4	MULTISPECIES: hypothetical protein	other
gene0856	Scaffold1	cluster4	MULTISPECIES: S8 family peptidase	biosynthetic-additional
gene0857	Scaffold1	cluster4	MULTISPECIES: hypothetical protein	other
gene0858	Scaffold1	cluster4	MULTISPECIES: poly-gamma-glutamate hydrolase family protein	other
gene0859	Scaffold1	cluster4	MULTISPECIES: OsmC family protein	other
gene0860	Scaffold1	cluster4	MULTISPECIES: multidrug efflux SMR transporter	other
gene0861	Scaffold1	cluster4	small multidrug resistance efflux transporter	other
gene0862	Scaffold1	cluster4	MULTISPECIES: hypothetical protein	other
gene0863	Scaffold1	cluster4	MULTISPECIES: YmaF family protein	other
gene0864	Scaffold1	cluster4	MULTISPECIES: tRNA (adenosine(37)-N6)-dimethylallyltransferase MiaA	other
gene0865	Scaffold1	cluster4	MULTISPECIES: RNA chaperone Hfq	other
gene0866	Scaffold1	cluster4	MULTISPECIES: YmzC family protein	other
gene0867	Scaffold1	cluster4	MULTISPECIES: hypothetical protein	other
gene0868	Scaffold1	cluster4	MULTISPECIES: class Ib ribonucleoside-diphosphate reductase assembly flavoprotein NrdI	other
gene0952	Scaffold1	cluster5	MULTISPECIES: sugar kinase	biosynthetic-additional
gene0953	Scaffold1	cluster5	MULTISPECIES: zinc-binding alcohol dehydrogenase family protein	biosynthetic-additional
gene0954	Scaffold1	cluster5	MULTISPECIES: bifunctional 2-keto-4-hydroxyglutarate aldolase/2-keto-3-deoxy-6-phosphogluconate aldolase	other
gene0955	Scaffold1	cluster5	MULTISPECIES: mannonate dehydratase	other
gene0956	Scaffold1	cluster5	MULTISPECIES: SDR family oxidoreductase	biosynthetic-additional
gene0957	Scaffold1	cluster5	MULTISPECIES: substrate-binding domain-containing protein	regulatory
gene0958	Scaffold1	cluster5	MULTISPECIES: MFS transporter	transport
gene0959	Scaffold1	cluster5	MULTISPECIES: hypothetical protein	other

gene0960	Scaffold1	cluster5	MULTISPECIES: DUF4166 domain-containing protein	other
gene0961	Scaffold1	cluster5	MULTISPECIES: YndJ family protein	other
gene0962	Scaffold1	cluster5	MULTISPECIES: choline esterase	other
gene0963	Scaffold1	cluster5	-	other
gene0964	Scaffold1	cluster5	MULTISPECIES: cellulase family glycosylhydrolase	other
gene0965	Scaffold1	cluster5	MULTISPECIES: DUF4870 domain-containing protein	other
gene0966	Scaffold1	cluster5	MULTISPECIES: HAMP domain-containing sensor histidine kinase	regulatory
gene0967	Scaffold1	cluster5	MULTISPECIES: response regulator transcription factor	regulatory
gene0968	Scaffold1	cluster5	MULTISPECIES: glycoside hydrolase family 30 beta sandwich domain-containing protein	other
gene0969	Scaffold1	cluster5	MULTISPECIES: glycoside hydrolase family 43 protein	other
gene0970	Scaffold1	cluster5	MULTISPECIES: non-ribosomal peptide synthetase	biosynthetic
gene0971	Scaffold1	cluster5	MULTISPECIES: non-ribosomal peptide synthetase	biosynthetic
gene0972	Scaffold1	cluster5	non-ribosomal peptide synthetase	biosynthetic
gene0973	Scaffold1	cluster5	MULTISPECIES: bacillomycin D biosynthesis malonyl-CoA transacylase BamD	biosynthetic
gene0974	Scaffold1	cluster5	MULTISPECIES: 3-hydroxybutyrate dehydrogenase	biosynthetic-additional
gene0975	Scaffold1	cluster5	MULTISPECIES: CoA transferase subunit B	other
gene0976	Scaffold1	cluster5	succinyl CoA:3-oxoacid CoA-transferase subunit A	other
gene0977	Scaffold1	cluster5	MULTISPECIES: GntP family permease	other
gene0978	Scaffold1	cluster5	MULTISPECIES: cytochrome P450	biosynthetic-additional
gene0979	Scaffold1	cluster5	MULTISPECIES: biotin synthase BioB	biosynthetic-additional
gene0980	Scaffold1	cluster5	MULTISPECIES: dethiobiotin synthase	other
gene0981	Scaffold1	cluster5	MULTISPECIES: 8-amino-7-oxononanoate synthase	biosynthetic-additional
gene0982	Scaffold1	cluster5	MULTISPECIES: adenosylmethionine--8-amino-7-oxononanoate transaminase	biosynthetic-additional
gene0983	Scaffold1	cluster5	MULTISPECIES: 6-carboxyhexanoate--CoA ligase	other
gene0984	Scaffold1	cluster5	MULTISPECIES: GtrA family protein	other
gene0985	Scaffold1	cluster5	MULTISPECIES: UTP--glucose-1-phosphate uridylyltransferase GalU	biosynthetic-additional
gene0986	Scaffold1	cluster5	MULTISPECIES: DedA family protein	other
gene0987	Scaffold1	cluster5	MULTISPECIES: acyl-CoA carboxylase subunit beta	biosynthetic-additional
gene0988	Scaffold1	cluster5	MULTISPECIES: enoyl-CoA hydratase	biosynthetic-additional
gene0989	Scaffold1	cluster5	MULTISPECIES: hydroxymethylglutaryl-CoA lyase	biosynthetic
gene0990	Scaffold1	cluster5	MULTISPECIES: acetyl-CoA carboxylase biotin carboxyl carrier protein subunit	other
gene0991	Scaffold1	cluster5	MULTISPECIES: acetyl-CoA carboxylase biotin carboxylase subunit	biosynthetic-additional
gene0992	Scaffold1	cluster5	MULTISPECIES: AMP-binding protein	biosynthetic
gene0993	Scaffold1	cluster5	MULTISPECIES: acyl-CoA dehydrogenase family protein	biosynthetic-additional
gene0994	Scaffold1	cluster5	MULTISPECIES: family 10 glycosylhydrolase	other
gene0995	Scaffold1	cluster5	MULTISPECIES: DUF1360 domain-containing protein	other
gene0996	Scaffold1	cluster5	MULTISPECIES: non-ribosomal peptide synthetase	biosynthetic

gene0997	Scaffold1	cluster5	non-ribosomal peptide synthetase	biosynthetic
gene0998	Scaffold1	cluster5	non-ribosomal peptide synthetase	biosynthetic
gene0999	Scaffold1	cluster5	MULTISPECIES: non-ribosomal peptide synthetase	biosynthetic
gene1000	Scaffold1	cluster5	MULTISPECIES: non-ribosomal peptide synthetase	biosynthetic
gene1001	Scaffold1	cluster5	non-ribosomal peptide synthetase	biosynthetic
gene1002	Scaffold1	cluster5	MULTISPECIES: D-alanyl-D-alanine carboxypeptidase/D-alanyl-D-alanine-endopeptidase	other
gene1003	Scaffold1	cluster5	MULTISPECIES: aldose 1-epimerase	other
gene1004	Scaffold1	cluster5	MULTISPECIES: MATE family efflux transporter	transport
gene1005	Scaffold1	cluster5	MULTISPECIES: IseA DL-endopeptidase inhibitor family protein	other
gene1007	Scaffold1	cluster5	MULTISPECIES: site-specific integrase	other
gene1008	Scaffold1	cluster5	MULTISPECIES: excisionase family DNA-binding protein	other
gene1009	Scaffold1	cluster5	condensation domain-containing protein	biosynthetic-additional
gene1010	Scaffold1	cluster5	non-ribosomal peptide synthetase	biosynthetic
gene1011	Scaffold1	cluster5	MULTISPECIES: excisionase family DNA-binding protein	other
gene1012	Scaffold1	cluster5	MULTISPECIES: gamma-glutamyltransferase	biosynthetic-additional
gene1013	Scaffold1	cluster5	MULTISPECIES: MFS transporter	transport
gene1014	Scaffold1	cluster5	MULTISPECIES: metalloregulator ArsR/SmtB family transcription factor	regulatory
gene1015	Scaffold1	cluster5	MULTISPECIES: His/Gly/Thr/Pro-type tRNA ligase C-terminal domain-containing protein	other
gene1016	Scaffold1	cluster5	MULTISPECIES: LysR family transcriptional regulator	regulatory
gene1017	Scaffold1	cluster5	MULTISPECIES: zinc-binding dehydrogenase	biosynthetic-additional
gene1018	Scaffold1	cluster5	MULTISPECIES: glutamate synthase small subunit	other
gene1019	Scaffold1	cluster5	MULTISPECIES: glutamate synthase large subunit	other
gene1020	Scaffold1	cluster5	LysR family transcriptional regulator	regulatory
gene1021	Scaffold1	cluster5	MULTISPECIES: glutamate 5-kinase	other
gene1022	Scaffold1	cluster5	pyrroline-5-carboxylate reductase	other
gene1023	Scaffold1	cluster5	MULTISPECIES: replication termination protein	other
gene1024	Scaffold1	cluster5	MULTISPECIES: 3-ketoacyl-ACP reductase	biosynthetic-additional
gene1025	Scaffold1	cluster5	MULTISPECIES: hypothetical protein	other
gene1026	Scaffold1	cluster5	MULTISPECIES: hypothetical protein	other
gene1040	Scaffold1	cluster6	MULTISPECIES: DUF4236 domain-containing protein	other
gene1041	Scaffold1	cluster6	MULTISPECIES: hypothetical protein	other
gene1042	Scaffold1	cluster6	MULTISPECIES: hypothetical protein	other
gene1043	Scaffold1	cluster6	MULTISPECIES: hypothetical protein	other
gene1044	Scaffold1	cluster6	hypothetical protein	other
gene1045	Scaffold1	cluster6	MULTISPECIES: hypothetical protein	other
gene1046	Scaffold1	cluster6	MULTISPECIES: DUF692 domain-containing protein	biosynthetic
gene1047	Scaffold1	cluster6	MULTISPECIES: MFS transporter	transport
gene1048	Scaffold1	cluster6	MULTISPECIES: hypothetical protein	other
gene1049	Scaffold1	cluster6	hypothetical protein	other
gene1050	Scaffold1	cluster6	MULTISPECIES: glucose 1-dehydrogenase	biosynthetic-additional
gene1051	Scaffold1	cluster6	MULTISPECIES: winged helix-turn-helix transcriptional	regulatory

			regulator	
gene1063	Scaffold1	cluster7	hypothetical protein	other
gene1064	Scaffold1	cluster7	MULTISPECIES: 3D domain-containing protein	other
gene1065	Scaffold1	cluster7	MULTISPECIES: DNA helicase RecQ	other
gene1066	Scaffold1	cluster7	MULTISPECIES: FMN-dependent NADH-azoreductase	other
gene1067	Scaffold1	cluster7	hypothetical protein	other
gene1068	Scaffold1	cluster7	MULTISPECIES: TraR/DksA C4-type zinc finger protein	other
gene1069	Scaffold1	cluster7	hypothetical protein	other
gene1070	Scaffold1	cluster7	MULTISPECIES: hypothetical protein	other
gene1071	Scaffold1	cluster7	MULTISPECIES: Hsp20/alpha crystallin family protein	other
gene1072	Scaffold1	cluster7	MULTISPECIES: hypothetical protein	other
gene1073	Scaffold1	cluster7	MULTISPECIES: hypothetical protein	other
gene1074	Scaffold1	cluster7	MULTISPECIES: PH domain-containing protein	other
gene1075	Scaffold1	cluster7	MULTISPECIES: DUF4025 domain-containing protein	other
gene1076	Scaffold1	cluster7	MULTISPECIES: DUF6501 family protein	other
gene1077	Scaffold1	cluster7	MULTISPECIES: aldehyde dehydrogenase family protein	biosynthetic-additional
gene1078	Scaffold1	cluster7	MULTISPECIES: prenyltransferase/squalene oxidase repeat-containing protein	biosynthetic
gene1079	Scaffold1	cluster7	MULTISPECIES: superoxide dismutase	other
gene1080	Scaffold1	cluster7	MULTISPECIES: sodium-dependent transporter	other
gene1081	Scaffold1	cluster7	bile acid:sodium symporter family protein	other
gene1082	Scaffold1	cluster7	MULTISPECIES: MBL fold metallo-hydrolase	biosynthetic-additional
gene1083	Scaffold1	cluster7	MULTISPECIES: 2-oxoglutarate dehydrogenase complex dihydrolipoyllysine-residue succinyltransferase	other
gene1084	Scaffold1	cluster7	MULTISPECIES: 2-oxoglutarate dehydrogenase E1 component	other
gene1174	Scaffold1	cluster8	MULTISPECIES: dihydrofolate reductase	other
gene1175	Scaffold1	cluster8	MULTISPECIES: thymidylate synthase	other
gene1176	Scaffold1	cluster8	MULTISPECIES: YpjP family protein	other
gene1177	Scaffold1	cluster8	class I SAM-dependent methyltransferase	other
gene1178	Scaffold1	cluster8	MULTISPECIES: BrxA/BrxB family bacilliredoxin	other
gene1179	Scaffold1	cluster8	MULTISPECIES: dihydroxy-acid dehydratase	other
gene1180	Scaffold1	cluster8	MULTISPECIES: conserved virulence factor C family protein	other
gene1181	Scaffold1	cluster8	MULTISPECIES: HD domain-containing protein	other
gene1182	Scaffold1	cluster8	MULTISPECIES: glutathione peroxidase	other
gene1183	Scaffold1	cluster8	Homoserine O-acetyltransferase	other
gene1184	Scaffold1	cluster8	MULTISPECIES: diglucosyl diacylglycerol synthase	biosynthetic-additional
gene1185	Scaffold1	cluster8	MULTISPECIES: cold-shock protein CspD	regulatory
gene1186	Scaffold1	cluster8	MULTISPECIES: hypothetical protein	other
gene1187	Scaffold1	cluster8	MULTISPECIES: DUF2564 family protein	other
gene1188	Scaffold1	cluster8	MULTISPECIES: zinc-finger domain-containing protein	other
gene1189	Scaffold1	cluster8	MULTISPECIES: ribonuclease H family protein	other
gene1190	Scaffold1	cluster8	MULTISPECIES: queuosine precursor transporter	other
gene1191	Scaffold1	cluster8	hypothetical protein	other
gene1192	Scaffold1	cluster8	MULTISPECIES: small, acid-soluble spore protein L	other
gene1193	Scaffold1	cluster8	MULTISPECIES: 5'-3' exonuclease	other
gene1194	Scaffold1	cluster8	MULTISPECIES: hypothetical protein	other
gene1195	Scaffold1	cluster8	MULTISPECIES: YpbS family protein	other

gene1196	Scaffold1	cluster8	MULTISPECIES: dynamin family protein	other
gene1197	Scaffold1	cluster8	MULTISPECIES: isoprenylcysteine carboxyl methyltransferase family protein	biosynthetic-additional
gene1198	Scaffold1	cluster8	MULTISPECIES: 3-oxoacyl-[acyl-carrier-protein] synthase III C-terminal domain-containing protein	biosynthetic
gene1199	Scaffold1	cluster8	MULTISPECIES: GNAT family protein	biosynthetic-additional
gene1200	Scaffold1	cluster8	hypothetical protein	other
gene1201	Scaffold1	cluster8	MULTISPECIES: hypothetical protein	other
gene1202	Scaffold1	cluster8	hypothetical protein	other
gene1203	Scaffold1	cluster8	uncharacterized membrane protein YcaP (DUF421 family)	other
gene1204	Scaffold1	cluster8	MULTISPECIES: cysteine hydrolase family protein	biosynthetic-additional
gene1205	Scaffold1	cluster8	MULTISPECIES: hypothetical protein	other
gene1206	Scaffold1	cluster8	MULTISPECIES: nucleobase:cation symporter-2 family protein	other
gene1207	Scaffold1	cluster8	MULTISPECIES: xanthine phosphoribosyltransferase	other
gene1208	Scaffold1	cluster8	MULTISPECIES: carboxypeptidase M32	other
gene1209	Scaffold1	cluster8	MULTISPECIES: ATP-dependent DNA helicase	other
gene1210	Scaffold1	cluster8	MULTISPECIES: hypothetical protein	other
gene1211	Scaffold1	cluster8	MULTISPECIES: YpzG family protein	other
gene1212	Scaffold1	cluster8	MULTISPECIES: class I SAM-dependent RNA methyltransferase	other
gene1213	Scaffold1	cluster8	MULTISPECIES: cell division regulator GpsB	other
gene1214	Scaffold1	cluster8	MULTISPECIES: DUF1273 domain-containing protein	other
gene1215	Scaffold1	cluster8	MULTISPECIES: spore coat protein CotD	other
gene1216	Scaffold1	cluster8	hypothetical protein	other
gene1217	Scaffold1	cluster8	MULTISPECIES: ribonuclease H-like domain-containing protein	other
gene1218	Scaffold1	cluster8	MULTISPECIES: DEAD/DEAH box helicase	other
gene1219	Scaffold1	cluster8	MULTISPECIES: PTS glucose transporter subunit IIA	other
gene1220	Scaffold1	cluster8	MULTISPECIES: hypothetical protein	other
gene1221	Scaffold1	cluster8	MULTISPECIES: YppG family protein	other
gene1222	Scaffold1	cluster8	MULTISPECIES: YppF family protein	other
gene1223	Scaffold1	cluster8	MULTISPECIES: YppE family protein	other
gene1375	Scaffold1	cluster9	MULTISPECIES: SDR family oxidoreductase	biosynthetic-additional
gene1376	Scaffold1	cluster9	MULTISPECIES: MBL fold metallo-hydrolase	biosynthetic-additional
gene1377	Scaffold1	cluster9	MULTISPECIES: pyrroline-5-carboxylate reductase	other
gene1378	Scaffold1	cluster9	MULTISPECIES: polyketide synthase	biosynthetic-additional
gene1379	Scaffold1	cluster9	difficidin synthesis, hydroxymethylglutaryl-CoA synthase	biosynthetic-additional
gene1380	Scaffold1	cluster9	cytochrome P450	biosynthetic-additional
gene1381	Scaffold1	cluster9	beta-ketoacyl synthase N-terminal-like domain-containing protein	biosynthetic
gene1382	Scaffold1	cluster9	type I polyketide synthase	biosynthetic
gene1383	Scaffold1	cluster9	type I polyketide synthase	biosynthetic
gene1384	Scaffold1	cluster9	SDR family NAD(P)-dependent oxidoreductase	biosynthetic
gene1385	Scaffold1	cluster9	MULTISPECIES: type I polyketide synthase	biosynthetic
gene1386	Scaffold1	cluster9	MULTISPECIES: beta-ketoacyl synthase N-terminal-like	biosynthetic

			domain-containing protein	
gene1387	Scaffold1	cluster9	MULTISPECIES: type I polyketide synthase	biosynthetic
gene1388	Scaffold1	cluster9	MULTISPECIES: SDR family oxidoreductase	biosynthetic-additional
gene1389	Scaffold1	cluster9	MULTISPECIES: fatty acid--CoA ligase family protein	biosynthetic-additional
gene1390	Scaffold1	cluster9	MULTISPECIES: acyl carrier protein	biosynthetic-additional
gene1391	Scaffold1	cluster9	MULTISPECIES: D-fructose-6-phosphate amidotransferase	other
gene1392	Scaffold1	cluster9	MULTISPECIES: ACP S-malonyltransferase	biosynthetic
gene1393	Scaffold1	cluster9	MULTISPECIES: antiterminator LoaP	other
gene1394	Scaffold1	cluster9	MULTISPECIES: LysR family transcriptional regulator	regulatory
gene1395	Scaffold1	cluster9	MULTISPECIES: GlpM family protein	other
gene1396	Scaffold1	cluster9	MULTISPECIES: NADPH dehydrogenase NamA	biosynthetic-additional
gene1397	Scaffold1	cluster9	MULTISPECIES: alpha/beta hydrolase	biosynthetic-additional
gene1398	Scaffold1	cluster9	MULTISPECIES: ribonuclease Z	other
gene1399	Scaffold1	cluster9	MULTISPECIES: glucose-6-phosphate dehydrogenase	other
gene1400	Scaffold1	cluster9	MULTISPECIES: NADP-dependent phosphogluconate dehydrogenase	biosynthetic-additional
gene1401	Scaffold1	cluster9	MULTISPECIES: DNA polymerase IV	other
gene1402	Scaffold1	cluster9	MULTISPECIES: hypothetical protein	other
gene1403	Scaffold1	cluster9	MULTISPECIES: membrane protein insertase YidC	other
gene1404	Scaffold1	cluster9	MULTISPECIES: tripeptidase T	biosynthetic-additional
gene1405	Scaffold1	cluster9	propionyl-CoA carboxylase subunit beta	biosynthetic-additional
gene1406	Scaffold1	cluster9	MULTISPECIES: methylmalonyl-CoA epimerase	other
gene1407	Scaffold1	cluster9	MULTISPECIES: stressosome-associated protein Prli42	other
gene1408	Scaffold1	cluster9	MULTISPECIES: L,D-transpeptidase	other
gene1409	Scaffold1	cluster9	MULTISPECIES: aromatic acid exporter family protein	other
gene1410	Scaffold1	cluster9	MULTISPECIES: amino acid ABC transporter ATP-binding protein	transport
gene1411	Scaffold1	cluster9	MULTISPECIES: amino acid ABC transporter permease	transport
gene1412	Scaffold1	cluster9	transporter substrate-binding domain-containing protein	biosynthetic-additional
gene1413	Scaffold1	cluster9	MULTISPECIES: bacilliredoxin BrxB	other
gene2064	Scaffold2	cluster10	Na(+)/H(+) antiporter subunit D	other
gene2065	Scaffold2	cluster10	MULTISPECIES: Na+/H+ antiporter subunit E	other
gene2066	Scaffold2	cluster10	MULTISPECIES: Na(+)/H(+) antiporter subunit F1	other
gene2067	Scaffold2	cluster10	MULTISPECIES: monovalent cation/H(+) antiporter subunit G	other
gene2068	Scaffold2	cluster10	MULTISPECIES: hotdog fold thioesterase	biosynthetic-additional
gene2069	Scaffold2	cluster10	MULTISPECIES: response regulator transcription factor	regulatory
gene2070	Scaffold2	cluster10	hypothetical protein	regulatory
gene2071	Scaffold2	cluster10	MULTISPECIES: histidine kinase	other
gene2072	Scaffold2	cluster10	MULTISPECIES: competence pheromone ComX	other
gene2073	Scaffold2	cluster10	competence protein ComQ	biosynthetic-additional
gene2074	Scaffold2	cluster10	MULTISPECIES: degradation enzyme regulation protein DegQ	other

gene2075	Scaffold2	cluster10	hypothetical protein	other
gene2076	Scaffold2	cluster10	MULTISPECIES: hypothetical protein	other
gene2077	Scaffold2	cluster10	MULTISPECIES: HDOD domain-containing protein	other
gene2078	Scaffold2	cluster10	MULTISPECIES: nicotinate phosphoribosyltransferase	other
gene2079	Scaffold2	cluster10	MULTISPECIES: isochorismatase family cysteine hydrolase	biosynthetic-additional
gene2080	Scaffold2	cluster10	MULTISPECIES: DUF1694 domain-containing protein	other
gene2081	Scaffold2	cluster10	MULTISPECIES: YueH family protein	other
gene2082	Scaffold2	cluster10	MULTISPECIES: spore germination protein	other
gene2083	Scaffold2	cluster10	pheromone autoinducer 2 transporter	other
gene2084	Scaffold2	cluster10	MULTISPECIES: DUF2642 domain-containing protein	other
gene2085	Scaffold2	cluster10	MULTISPECIES: HD domain-containing protein	other
gene2086	Scaffold2	cluster10	MULTISPECIES: (S)-benzoin forming benzil reductase	biosynthetic-additional
gene2087	Scaffold2	cluster10	MULTISPECIES: PucR family transcriptional regulator	other
gene2088	Scaffold2	cluster10	MULTISPECIES: alanine dehydrogenase	other
gene2089	Scaffold2	cluster10	MULTISPECIES: YukJ family protein	other
gene2090	Scaffold2	cluster10	MULTISPECIES: MbtH family protein	biosynthetic-additional
gene2091	Scaffold2	cluster10	MULTISPECIES: non-ribosomal peptide synthetase	biosynthetic
gene2092	Scaffold2	cluster10	MULTISPECIES: isochorismatase	biosynthetic-additional
gene2093	Scaffold2	cluster10	MULTISPECIES: (2,3-dihydroxybenzoyl)adenylate synthase	biosynthetic-additional
gene2094	Scaffold2	cluster10	MULTISPECIES: isochorismate synthase DhbC	biosynthetic-additional
gene2095	Scaffold2	cluster10	MULTISPECIES: 2,3-dihydro-2,3-dihydroxybenzoate dehydrogenase	biosynthetic-additional
gene2096	Scaffold2	cluster10	MULTISPECIES: alpha/beta hydrolase	biosynthetic-additional
gene2097	Scaffold2	cluster10	MULTISPECIES: sulfite oxidase-like oxidoreductase	other
gene2098	Scaffold2	cluster10	MULTISPECIES: biotin transporter BioY	other
gene2099	Scaffold2	cluster10	MULTISPECIES: SLC13 family permease	other
gene2100	Scaffold2	cluster10	MULTISPECIES: leucyl aminopeptidase	other
gene2101	Scaffold2	cluster10	MULTISPECIES: divergent PAP2 family protein	other
gene2102	Scaffold2	cluster10	MULTISPECIES: 3D domain-containing protein	other
gene2103	Scaffold2	cluster10	YuiB family protein	other
gene2104	Scaffold2	cluster10	MULTISPECIES: NAD(P)/FAD-dependent oxidoreductase	biosynthetic-additional
gene2105	Scaffold2	cluster10	MULTISPECIES: ferredoxin--NADP reductase 2	biosynthetic-additional
gene2106	Scaffold2	cluster10	MULTISPECIES: hypothetical protein	other
gene2107	Scaffold2	cluster10	MULTISPECIES: GMP reductase	other
gene2108	Scaffold2	cluster10	MULTISPECIES: DUF5970 family protein	other
gene2109	Scaffold2	cluster10	hypothetical protein	other
gene2110	Scaffold2	cluster10	MULTISPECIES: ABC transporter ATP-binding protein	transport
gene2111	Scaffold2	cluster10	MULTISPECIES: hypothetical protein	other
gene2112	Scaffold2	cluster10	hypothetical protein	biosynthetic
gene2113	Scaffold2	cluster10	MULTISPECIES: hypothetical protein	other
gene2114	Scaffold2	cluster10	MULTISPECIES: MerR family transcriptional regulator	other

gene2115	Scaffold2	cluster10	MULTISPECIES: SDR family NAD(P)-dependent oxidoreductase	biosynthetic-additional
gene2116	Scaffold2	cluster10	MULTISPECIES: iron-sulfur cluster assembly accessory protein	other
gene2117	Scaffold2	cluster10	MULTISPECIES: diaminopimelate epimerase	other
gene2118	Scaffold2	cluster10	MULTISPECIES: NupC/NupG family nucleoside CNT transporter	other
gene2642	Scaffold2	cluster11	MULTISPECIES: transglycosylase domain-containing protein	other
gene2643	Scaffold2	cluster11	MULTISPECIES: hypothetical protein	other
gene2644	Scaffold2	cluster11	MULTISPECIES: YwhD family protein	other
gene2645	Scaffold2	cluster11	MULTISPECIES: site-2 protease family protein	biosynthetic-additional
gene2646	Scaffold2	cluster11	MULTISPECIES: 2-hydroxymuconate tautomerase	other
gene2647	Scaffold2	cluster11	MULTISPECIES: MarR family transcriptional regulator	regulatory
gene2648	Scaffold2	cluster11	putative amino acid permease YhdG	biosynthetic-additional
gene2649	Scaffold2	cluster11	MULTISPECIES: YwgA family protein	other
gene2650	Scaffold2	cluster11	MULTISPECIES: HD domain-containing protein	other
gene2651	Scaffold2	cluster11	MULTISPECIES: DUF1450 domain-containing protein	other
gene2652	Scaffold2	cluster11	MULTISPECIES: RsfA family transcriptional regulator	other
gene2653	Scaffold2	cluster11	MULTISPECIES: PadR family transcriptional regulator	other
gene2654	Scaffold2	cluster11	MULTISPECIES: DUF1700 domain-containing protein	other
gene2655	Scaffold2	cluster11	MULTISPECIES: biotin/lipoate A/B protein ligase family protein	other
gene2656	Scaffold2	cluster11	MULTISPECIES: LysR family transcriptional regulator	regulatory
gene2657	Scaffold2	cluster11	hypothetical protein	other
gene2658	Scaffold2	cluster11	MULTISPECIES: phosphate acetyltransferase	other
gene2659	Scaffold2	cluster11	MULTISPECIES: heme-dependent peroxidase	other
gene2660	Scaffold2	cluster11	NADPH-dependent reductase BacG	biosynthetic-additional
gene2661	Scaffold2	cluster11	MULTISPECIES: pyridoxal phosphate-dependent aminotransferase	biosynthetic-additional
gene2662	Scaffold2	cluster11	MULTISPECIES: MFS transporter	transport
gene2663	Scaffold2	cluster11	MULTISPECIES: ATP-grasp domain-containing protein	biosynthetic
gene2664	Scaffold2	cluster11	MULTISPECIES: dihydroanticapsin 7-dehydrogenase	biosynthetic-additional
gene2665	Scaffold2	cluster11	MULTISPECIES: cupin domain-containing protein	other
gene2666	Scaffold2	cluster11	MULTISPECIES: bacilysin biosynthesis protein BacA	other
gene2667	Scaffold2	cluster11	MULTISPECIES: MFS transporter	transport
gene2668	Scaffold2	cluster11	MULTISPECIES: multidrug effflux MFS transporter	transport
gene2669	Scaffold2	cluster11	MULTISPECIES: amino acid permease	biosynthetic-additional
gene2670	Scaffold2	cluster11	MULTISPECIES: M20/M25/M40 family metallo-hydrolase	other
gene2671	Scaffold2	cluster11	MULTISPECIES: L-glutamate gamma-semialdehyde dehydrogenase	biosynthetic-additional
gene2672	Scaffold2	cluster11	MULTISPECIES: Glu/Leu/Phe/Val dehydrogenase	other
gene2673	Scaffold2	cluster11	MULTISPECIES: biofilm surface layer hydrophobin BslB	other
gene2674	Scaffold2	cluster11	MULTISPECIES: dTDP-4-dehydrorhamnose 3,5-epimerase family protein	biosynthetic-additional
gene2675	Scaffold2	cluster11	MULTISPECIES: dTDP-4-dehydrorhamnose reductase	biosynthetic-additional
gene2676	Scaffold2	cluster11	MULTISPECIES: dTDP-glucose 4,6-dehydratase	biosynthetic-add

				itional
gene2677	Scaffold2	cluster11	MULTISPECIES: sugar phosphate nucleotidyltransferase	biosynthetic-additional
gene2678	Scaffold2	cluster11	MULTISPECIES: spore coat protein	biosynthetic-additional
gene2679	Scaffold2	cluster11	spore coat protein	other
gene2680	Scaffold2	cluster11	MULTISPECIES: N-acetylneuraminate synthase family protein	other
gene2681	Scaffold2	cluster11	MULTISPECIES: GNAT family N-acetyltransferase	other
gene2682	Scaffold2	cluster11	MULTISPECIES: DegT/DnrJ/EryC1/StrS family aminotransferase	biosynthetic-additional
gene2827	Scaffold2	cluster12	MULTISPECIES: ABC transporter ATP-binding protein YxdL	transport
gene2828	Scaffold2	cluster12	MULTISPECIES: two-component system sensor histidine kinase YxdK	regulatory
gene2829	Scaffold2	cluster12	MULTISPECIES: two-component system response regulator YxdJ	regulatory
gene2830	Scaffold2	cluster12	MULTISPECIES: HAMP domain-containing sensor histidine kinase	regulatory
gene2831	Scaffold2	cluster12	MULTISPECIES: response regulator transcription factor	regulatory
gene2832	Scaffold2	cluster12	MULTISPECIES: ABC transporter ATP-binding protein	transport
gene2833	Scaffold2	cluster12	MULTISPECIES: ABC transporter permease	other
gene2834	Scaffold2	cluster12	MULTISPECIES: ABC transporter permease	other
gene2835	Scaffold2	cluster12	MULTISPECIES: plantaricin C family lantibiotic	biosynthetic-additional
gene2836	Scaffold2	cluster12	MULTISPECIES: winged helix-turn-helix domain-containing protein	other
gene2837	Scaffold2	cluster12	MULTISPECIES: flavoprotein	biosynthetic-additional
gene2838	Scaffold2	cluster12	MULTISPECIES: type 2 lanthipeptide synthetase LanM family protein	biosynthetic
gene2839	Scaffold2	cluster12	MULTISPECIES: peptidase domain-containing ABC transporter	transport
gene2840	Scaffold2	cluster12	class II fructose-1,6-bisphosphate aldolase	other
gene2841	Scaffold2	cluster12	MULTISPECIES: 2-keto-myo-inositol isomerase	other
gene2842	Scaffold2	cluster12	MULTISPECIES: sugar phosphate isomerase/epimerase	other
gene2843	Scaffold2	cluster12	MULTISPECIES: Gfo/Idh/MocA family oxidoreductase	biosynthetic-additional
gene2844	Scaffold2	cluster12	MFS transporter	transport
gene2845	Scaffold2	cluster12	MULTISPECIES: myo-inosose-2 dehydratase	other
gene3057	Scaffold3	cluster13	DNA ligase	other
gene3058	Scaffold3	cluster13	MULTISPECIES: CamS family sex pheromone protein	other
gene3059	Scaffold3	cluster13	MULTISPECIES: phosphotransferase enzyme family protein	biosynthetic-additional
gene3060	Scaffold3	cluster13	MULTISPECIES: MgtC/SapB family protein	other
gene3061	Scaffold3	cluster13	MULTISPECIES: sodium/proline symporter PutP	other
gene3062	Scaffold3	cluster13	MULTISPECIES: Asp-tRNA(Asn)/Glu-tRNA(Gln) amidotransferase subunit GatC	other
gene3063	Scaffold3	cluster13	MULTISPECIES: Asp-tRNA(Asn)/Glu-tRNA(Gln) amidotransferase subunit GatA	biosynthetic-additional
gene3064	Scaffold3	cluster13	MULTISPECIES: Asp-tRNA(Asn)/Glu-tRNA(Gln) amidotransferase subunit GatB	other
gene3065	Scaffold3	cluster13	MULTISPECIES: TetR/AcrR family transcriptional regulator	regulatory
gene3066	Scaffold3	cluster13	MULTISPECIES: efflux RND transporter permease subunit	transport

gene3067	Scaffold3	cluster13	MULTISPECIES: diacylglycerol kinase	other
gene3068	Scaffold3	cluster13	MULTISPECIES: 23S rRNA (uracil(1939)-C(5))-methyltransferase RlmD	other
gene3069	Scaffold3	cluster13	MULTISPECIES: response regulator transcription factor	regulatory
gene3070	Scaffold3	cluster13	MULTISPECIES: hypothetical protein	other
gene3071	Scaffold3	cluster13	acyl carrier protein	biosynthetic-additional
gene3072	Scaffold3	cluster13	MULTISPECIES: 3-hydroxyacyl-ACP dehydratase FabZ	biosynthetic-additional
gene3073	Scaffold3	cluster13	3-oxoacyl-ACP synthase	biosynthetic
gene3074	Scaffold3	cluster13	MULTISPECIES: beta-ketoacyl synthase N-terminal-like domain-containing protein	biosynthetic-additional
gene3075	Scaffold3	cluster13	MULTISPECIES: SDR family oxidoreductase	biosynthetic-additional
gene3076	Scaffold3	cluster13	MULTISPECIES: ATP-binding cassette domain-containing protein	transport
gene3077	Scaffold3	cluster13	MULTISPECIES: ABC transporter permease	other
gene3078	Scaffold3	cluster13	MULTISPECIES: antitoxin YezG family protein	other
gene3079	Scaffold3	cluster13	MULTISPECIES: DUF600 family protein	other
gene3080	Scaffold3	cluster13	MULTISPECIES: YebC/PmpR family DNA-binding transcriptional regulator	other
gene3081	Scaffold3	cluster13	hypothetical protein	other
gene3082	Scaffold3	cluster13	MULTISPECIES: aminoglycoside 6-adenylyltransferase	other
gene3083	Scaffold3	cluster13	MULTISPECIES: spore coat-associated protein CotJA	other
gene3084	Scaffold3	cluster13	MULTISPECIES: spore coat protein CotJB	other
gene3085	Scaffold3	cluster13	MULTISPECIES: spore coat protein CotJC	other
gene3086	Scaffold3	cluster13	MULTISPECIES: GNAT family N-acetyltransferase	other
gene3087	Scaffold3	cluster13	MULTISPECIES: YesK-like family protein	other
gene3088	Scaffold3	cluster13	MULTISPECIES: DUF421 domain-containing protein	other
gene3089	Scaffold3	cluster13	MULTISPECIES: heme-degrading oxygenase HmoA	other
gene3090	Scaffold3	cluster13	hypothetical protein	other
gene3091	Scaffold3	cluster13	MULTISPECIES: STAS domain-containing protein	other
gene3092	Scaffold3	cluster13	MULTISPECIES: YezD family protein	other
gene3093	Scaffold3	cluster13	MULTISPECIES: hypothetical protein	other
gene3094	Scaffold3	cluster13	MULTISPECIES: Bax inhibitor-1 family protein	other
gene3095	Scaffold3	cluster13	MULTISPECIES: hypothetical protein	other
gene3096	Scaffold3	cluster13	MULTISPECIES: MarR family transcriptional regulator	regulatory
gene3097	Scaffold3	cluster13	MULTISPECIES: FAD-dependent oxidoreductase	other
gene3098	Scaffold3	cluster13	MULTISPECIES: DUF3900 domain-containing protein	other
gene3467	Scaffold4	cluster14	MULTISPECIES: GNAT family N-acetyltransferase	other
gene3468	Scaffold4	cluster14	MULTISPECIES: LLM class flavin-dependent oxidoreductase	biosynthetic-additional
gene3469	Scaffold4	cluster14	MULTISPECIES: GerAB/ArcD/ProY family transporter	other
gene3470	Scaffold4	cluster14	MULTISPECIES: Ger(x)C family spore germination protein	other
gene3471	Scaffold4	cluster14	MULTISPECIES: spore germination protein	other
gene3472	Scaffold4	cluster14	MULTISPECIES: hypothetical protein	other
gene3473	Scaffold4	cluster14	MULTISPECIES: right-handed parallel beta-helix repeat-containing protein	other
gene3474	Scaffold4	cluster14	MULTISPECIES: peptide MFS transporter	transport
gene3475	Scaffold4	cluster14	MULTISPECIES: hypothetical protein	other

gene3476	Scaffold4	cluster14	MULTISPECIES: non-oxidative hydroxyarylic acid decarboxylases subunit D	other
gene3477	Scaffold4	cluster14	MULTISPECIES: phenolic acid decarboxylase BsdC	other
gene3478	Scaffold4	cluster14	aromatic acid decarboxylase	biosynthetic-additional
gene3479	Scaffold4	cluster14	MULTISPECIES: LysR family transcriptional regulator	regulatory
gene3480	Scaffold4	cluster14	MULTISPECIES: cystine ABC transporter substrate-binding lipoprotein TcyA	biosynthetic-additional
gene3481	Scaffold4	cluster14	polar amino acid transport system permease protein	transport
gene3482	Scaffold4	cluster14	MULTISPECIES: cystine ABC transporter ATP-binding protein TcyC	transport
gene3483	Scaffold4	cluster14	MULTISPECIES: YitT family protein	other
gene3484	Scaffold4	cluster14	MULTISPECIES: 4'-phosphopantetheinyl transferase superfamily protein	biosynthetic-additional
gene3485	Scaffold4	cluster14	MULTISPECIES: aminotransferase class I/II-fold pyridoxal phosphate-dependent enzyme	biosynthetic-additional
gene3486	Scaffold4	cluster14	MULTISPECIES: surfactin biosynthesis thioesterase SrfAD	biosynthetic-additional
gene3487	Scaffold4	cluster14	MULTISPECIES: non-ribosomal peptide synthetase	biosynthetic
gene3488	Scaffold4	cluster14	non-ribosomal peptide synthetase	biosynthetic-additional
gene3489	Scaffold5	cluster15	surfactin non-ribosomal peptide synthetase SrfAA	biosynthetic
gene3490	Scaffold5	cluster15	MULTISPECIES: helix-turn-helix domain-containing protein	regulatory
gene3491	Scaffold5	cluster15	MULTISPECIES: 3-hexulose-6-phosphate synthase	other
gene3492	Scaffold5	cluster15	MULTISPECIES: 6-phospho-3-hexuloisomerase	other
gene3493	Scaffold5	cluster15	MULTISPECIES: NucA/NucB deoxyribonuclease domain-containing protein	other
gene3494	Scaffold5	cluster15	MULTISPECIES: competence protein ComJ	other
gene3495	Scaffold5	cluster15	Aryl-phospho-beta-D-glucosidase BglC	other
gene3496	Scaffold5	cluster15	MULTISPECIES: family 1 glycosylhydrolase	other
gene3497	Scaffold5	cluster15	MULTISPECIES: YckD family protein	other
gene3498	Scaffold5	cluster15	MULTISPECIES: RDD family protein	other
gene3499	Scaffold5	cluster15	MULTISPECIES: zinc-dependent alcohol dehydrogenase	biosynthetic-additional
gene3500	Scaffold5	cluster15	MULTISPECIES: GTP-binding protein	biosynthetic-additional
gene3501	Scaffold5	cluster15	MULTISPECIES: NADPH-nitrite reductase	biosynthetic-additional
gene3502	Scaffold5	cluster15	MULTISPECIES: nitrite reductase small subunit NirD	other
gene3503	Scaffold5	cluster15	MULTISPECIES: uroporphyrinogen-III C-methyltransferase	biosynthetic-additional
gene3504	Scaffold5	cluster15	MULTISPECIES: NAD(P)/FAD-dependent oxidoreductase	biosynthetic-additional
gene3505	Scaffold5	cluster15	MULTISPECIES: PucR family transcriptional regulator	other
gene3506	Scaffold5	cluster15	proline:sodium symporter PutP	other
gene3507	Scaffold5	cluster15	MULTISPECIES: L-glutamate gamma-semialdehyde dehydrogenase	biosynthetic-additional
gene3508	Scaffold5	cluster15	MULTISPECIES: proline dehydrogenase	other
gene3509	Scaffold5	cluster15	MULTISPECIES: nucleotidyltransferase domain-containing protein	other
gene3714	Scaffold6	cluster16	MULTISPECIES: glutamate--tRNA ligase	other
gene3715	Scaffold6	cluster16	MULTISPECIES: 2-C-methyl-D-erythritol 2,4-cyclodiphosphate	other

			synthase	
gene3716	Scaffold6	cluster16	MULTISPECIES: 2-C-methyl-D-erythritol 4-phosphate cytidylyltransferase	other
gene3717	Scaffold6	cluster16	MULTISPECIES: PIN/TRAM domain-containing protein	other
gene3718	Scaffold6	cluster16	MULTISPECIES: DNA integrity scanning diadenylate cyclase DisA	other
gene3719	Scaffold6	cluster16	MULTISPECIES: DNA repair protein RadA	other
gene3720	Scaffold6	cluster16	MULTISPECIES: S8 family serine peptidase	biosynthetic-additional
gene3721	Scaffold6	cluster16	MULTISPECIES: ABC transporter ATP-binding protein	transport
gene3722	Scaffold6	cluster16	MULTISPECIES: type 2 lanthipeptide synthetase LanM	biosynthetic
gene3723	Scaffold6	cluster16	MULTISPECIES: mersacidin/lichenicidin family type 2 lantibiotic	biosynthetic-additional
gene3724	Scaffold6	cluster16	MULTISPECIES: ATP-dependent protease ATP-binding subunit ClpC	other
gene3725	Scaffold6	cluster16	MULTISPECIES: protein arginine kinase	other
gene3726	Scaffold6	cluster16	activator of protein kinase McsB	other
gene3727	Scaffold6	cluster16	MULTISPECIES: transcriptional regulator CtsR	other
gene3807	Scaffold9	cluster17	hypothetical protein	biosynthetic-additional
gene3808	Scaffold9	cluster17	non-ribosomal peptide synthetase	biosynthetic
gene3809	Scaffold9	cluster17	MULTISPECIES: surfactin non-ribosomal peptide synthetase SrfAA	biosynthetic-additional

Notes: Biosynthetic gene clusters potentially involved in antimicrobial metabolite production were mined by using antiSMASH 7.0 (Blin et al., 2023). A total of 17 biosynthetic gene clusters (BGCs) of secondary metabolites were predicted in the genome of *B. velezensis* FJAT-57093.

Blin K, Shaw S, Augustijn HE, Reitz ZL, Biermann F, Alanjary M, Fetter A, Terlouw BR, Metcalf WW, Helfrich EJN, van Wezel GP, Medema MH, Weber T. antiSMASH 7.0: new and improved predictions for detection, regulation, chemical structures and visualisation. Nucleic Acids Res, 2023, 51(W1):W46-W50.
